# Early Phase Dose‐Finding Designs for CAR‐T Cell Therapies

**DOI:** 10.1002/pst.70102

**Published:** 2026-06-18

**Authors:** Weishi Chen, Pavel Mozgunov, Jimmy Mullaert, Xavier Paoletti

**Affiliations:** ^1^ MRC Biostatistics Unit University of Cambridge Cambridge UK; ^2^ Clinical Research Division Institut Curie Paris France; ^3^ University of Versaille St Quentin/Paris Saclay Paris France; ^4^ STAMPM, INSERM U1331 Paris France

**Keywords:** CAR‐T cells, dose finding, phase I clinical trials

## Abstract

Chimeric Antigen Receptor (CAR)‐T cell is an immunotherapy which revolutionised the treatment of relapsed/refractory lymphoma and leukaemia. It is shown to have a higher response rate, higher mid‐to‐long term overall survival, and lower toxicity than standard treatments. However, due to a lack of dose‐limiting toxicity (DLT) and unclear dose‐effect relationship, traditional phase I designs of clinical trials cannot lead to accurate selections of the optimal dose (OD). Beside clinical outcomes, the CAR‐T cell expansion from serial blood samples is measured at various time points. We propose a novel early phase dose‐finding design for CAR‐T cells, using both toxicity and activity endpoints to locate the OD. The number of CAR‐T cells measured in the peripheral blood is used to indicate activity, which is more sensitive than the short‐term clinical responses traditionally used. A Bi‐Exponential model is used for the repeated measures of the number of cells for each patient, and is estimated under a Bayesian framework. The model is motivated by biological concerns and is flexible enough to accommodate different shapes of the cell‐expansion curve. Three criteria for activity are considered: (1) the number of cells at specific time points, (2) the duration before all cells are eliminated, (3) the area under the cell‐expansion curve. Simulation studies show that the OD can be selected with high accuracy even under small sample sizes.

## 1 Introduction

Chimeric Antigen Receptor (CAR)‐T cells are engineered immune effector cells with defined specificity that can kill tumour cells as well as enhance immune surveillance to prevent tumour recurrence [1]. So far, around 300 CAR‐T cell clinical trials have been conducted worldwide [2]. CAR‐T cell therapies generally lead to higher objective and complete response rates, longer survival, as well as lower toxicities compared to standard treatments. It has recently been approved by the Food and Drug Administration (FDA) [3] and has completely revolutionised the treatment of relapsed/refractory lymphoma and leukaemia since then.

In Phase I clinical trials of new treatments, it is typically assumed that both the efficacy and the toxicity increase monotonically with the dose level. Hence, the aim of phase I dose‐finding trials is to find the maximum dose level within a target toxicity probability, which is referred to as the maximum tolerated dose (MTD), and the target toxicity is called the target toxicity level (TTL). However, no clear relationship between dose of CAR‐T cells (i.e., number of infused cells per kg) and antitumor activity can be detected from the reported trials so far. Rotte et al. [4] reviewed 39 trials on CAR‐T cells between 2010 and 2022 with at least two dose levels, 26 of them reported no dose‐activity relationship, and the remaining 13 reported positive relationship only among the lower doses and plateau among doses. Furthermore, due to lack of DLT being observed in trials with CAR‐T cells, the relationship between dose level and toxicity is also unclear. Consequently, the relationship between the MTD and the optimal dose (OD), the most active dose level with acceptable toxicity, is not known for most CAR‐T cells. Hence, applying classic designs of phase I dose‐finding trials might not be appropriate in this case.

Among CAR‐T trials being reported, the designs of phase I clinical trials are frequently unclear. In most cases, the 3 + 3 design [5], or designs adapted from it, are used, and the highest dose among the levels pre‐specified is most often selected due to no DLT [6, 7]. Even if a few DLTs have been reported, often cytokin release syndrome (CRS), this adverse event is not clearly dose‐dependent. However, as CAR‐T cells are expensive treatments, it is important that more efficient designs of phase I clinical trials are used. Among the FDA‐approved CAR‐T cell products, the typical price is around $424,000 [8].

CAR‐T cells are living compounds and infused cells may rapidly proliferate; this expansion exhausts after a few weeks and the cells die out (typically after 2 to 4 months). The expansion from serial blood samples can be measured at various time points for each patient [9]. According to Rotte et al. [4], there is little association between dose levels and clinical outcomes, but a positive association between dose levels and CAR‐T cell expansions in the peripheral blood, which suggests that the latter can be a more sensitive measure of biological activity than the clinical outcomes, and thus may be used as an efficacy endpoint in clinical trials.

There are many dose‐finding designs that use toxicity‐efficacy information in the literature, O'Quigley et al. [10], Thall and Cook [11], Takeda et al. [12], Lin et al. [13] among others that allow only binary or discrete efficacy endpoints, Bekele and Shen [14], Yeung et al. [15] among others emphasise the importance of using continuous efficacy endpoints; however, they only assume a single measurement of efficacy. Altzerinakou and Paoletti [16] propose a joint model for discrete time‐to‐event toxicity endpoint and repeated continuous biomarker that takes time into account for the efficacy endpoint, but the trajectory assumed for the biomarker does not allow for initial expansions, which only happen to living compounds. Hence, none of those designs allow direct inclusion of the CAR‐T expansion information.

Meanwhile, there is an increasing number of designs that incorporate pharmacokinetics/pharmacodynamics (PK/PD) measurements into dose‐finding designs. Yuan et al. [17] integrate patient‐level PK/PD information with toxicity and efficacy outcomes in a seamless Phase I/II design; Micallef et al. [18] developed an exposure‐driven EWOC that couples a population PK model with Bayesian logistic regression for dose escalation; Pantoja et al. [19] proposed dose‐exposure‐toxicity models that use PK exposure as a continuous metric, among others. The use of CAR‐T cell expansion can be considered as a PD measurement, as it is a reflection of how the infused CAR‐T cells interact with the human body. However, the aforementioned models for PK/PD information cannot be directly applied due to two main reasons. Firstly, the initial proliferation period of CAR‐T cell expansions needs to be reflected in the statistical model. However, most PK/PD models in the literature can only model the decay period of the drug, and thus would not be suitable for CAR‐T cells. Secondly, for traditional drugs, the area under the PK‐time curve (AUC) is usually an effective summary of the patient‐level PK/PD information. However, for CAR‐T cells, there is no clear summary of the CAR‐T cell expansion trajectories. The level of expansion that can guarantee the effectiveness of the treatment is not well‐defined. Hence, models specifically for the CAR‐T expansion and criteria for activities need to be developed.

The performance of dose‐finding designs is evaluated by simulations based on various scenarios of true toxicity/activity, which are often chosen subjectively. Therefore, there is always the possibility to artificially make the proposed design perform well by choosing easy scenarios. To remove this subjectivity, O'Quigley et al. [20] developed the evaluation tool, known as the non‐parametric benchmark, which gives an upper bound on the proportion of correct selection (PCS) of the OD under each scenario, regardless of the design. The original benchmark assumes a single agent with a binary toxicity endpoint, and it has been extended to other settings including combinations of agents with binary toxicity endpoint [21] and a single agent with continuous toxicity endpoints [22].

In this paper, we propose an early phase design for CAR‐T cell therapies that uses both the toxicity and the efficacy endpoints to locate the optimal dose level (OD). Using the DLT as a binary toxicity endpoint gives a set of safe doses, among which the most efficacious level will be selected. The rest of the paper will be structured as follows. Section 2 introduces the real trial this work is motivated by. Then, Section 3 gives the statistical models being proposed for toxicity and efficacy. The design is evaluated in Section 4 under extensive simulation studies and the results are compared to the benchmark we develop specifically for CAR‐T cells. We conclude in Section 5.

## 2 Motivating Trial

Primary central nervous system lymphoma (PCNSL) is an aggressive form of diffuse large B‐cell lymphoma (DLBCL) that exclusively localises in the central nervous system (CNS). A Phase I/II first‐in‐human study evaluating anti‐CD19‐CAR‐T cells (academic manufacturing), 28z1XX‐SUV39H1 KO (gene editing by CRISPR‐Cas9) is being designed in this disease. Anti‐CD19‐CAR‐T cells have demonstrated anti‐tumour activity associated with some levels of toxicity, but no dose‐toxicity relationship. No MTD has been reached so far for Anti‐CD19‐CAR‐T cells (without the knock out gene).

The primary objective is then to identify a safe and active dose of this new adoptive therapy; we selected the range of doses that had been tested in the Anti‐CD19‐CAR‐T cells therapy trial. Co‐primary endpoints include the risk of severe toxicity (i.e., grade 3 or 4 non haematological toxicity with a particular focus on grade 2 cytokine release syndrome) and the activity. The MTD is the dose whose risk of DLT is about 20%. Even though the clinical activity is of upmost interest, the recommended phase II level will be defined based on biological activity measured by CAR‐T cells expansion in the bloodstream. It is hypothesised that (i) absence of expansion is associated with low clinical activity and (ii) the dose with the strongest expansion level is associated with the best potential clinical activity. In DBLC, a maximum peak in the number of CAR‐T cells is expected between 9 and 12 days. CAR‐T cells will be titrated at Day 0, 1, 3, 7, 10, 14, 21, 28, and twice a month for 3 months. Two endpoints will be computed at the patient level: the highest number of CAR‐T value and the area under the time‐expansion curve.

The starting dose is $25\times 10^{6}$ CAR‐T cells infusion. Three dose levels will be explored. In the event of a plateau in the relationship between biological activity and dose, the lowest dose among the safe doses at the edge of the plateau will be selected for phase II trials.

## 3 Statistical Methodology

This section starts by introducing the CAR‐T cells data in Section 3.1. Then, the statistical models for activity and toxicity are presented in Sections 3.2 and 3.3, respectively. The proposed design is summarised in Section 3.4, and the model estimation method is shown in Section 3.5.

### 3.1 CAR‐T Cells Data

The CAR‐T cells data consists of a binary DLT outcome and repeated measurements of the continuous CAR‐T cells expansion, indicating the activity of this living treatment. For CAR‐T cells, most of the DLTs exhibit in the form of cytokine release syndrome (CRS), which usually happens within 10 days after infusion, and thus the DLT outcome is taken as binary. The CAR‐T cells expansion will be monitored for 3–4 months, and repeated measurements will be taken at pre‐defined times, common to all patients. Both the DLT and activity outcomes depend on the dose level, which is defined as the number of CAR‐T cells being infused per kilogramme of bodyweight. There are two main profiles of CAR‐T cell evolution that are of most interest to the clinicians: the infused cells exhaust and are progressively eliminated from the blood, or they proliferate and are maintained over some duration before being eliminated. In terms of the trajectories of the CAR‐T cells expansions over time, the former profile corresponds to a straight decreasing, and the latter corresponds to an increasing before decreasing.

**FIGURE 1:**
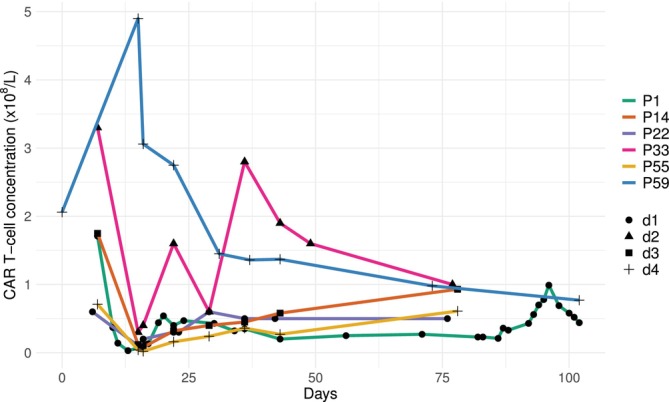
Trajectories of CAR‐T expansions reported by Cwynarski et al. [23]. Colours correspond to patients and point types correspond to the 4 dose levels being investigated.

As an example, selected CAR‐T cells data reported in Cwynarski et al. [23] are plotted in Figure 1, where the CAR‐T cells expansions of each patient are measured repeatedly over the next 3–4 months upon infusion of CAR‐T cells on day 0. The trajectory of patient 1 decreases straight after infusion, which exemplifies the former type, whereas the trajectory of patient 59 increases from the infused $2\times 10^{8}$ to $5\times 10^{8}$ cells per litre before decreasing towards zero, which exemplifies the latter type. Note that higher dose levels does not guarantee the increasing pattern, for example, patient 55 also receives the highest dose level $d_{4}$ but its trajectory decreases straight after infusion. Missing measurements are expected, for example, at day 0, only patient 59 had a recorded measurement. Moreover, not all trajectories fall into the two expected types, such as patient 33. If the efficacy model is strongly misspecified, the estimates for patient 33 will be biassed. This will be discussed at the end of this manuscript.

Motivated by the observed data, models for CAR‐T cell expansions should be flexible enough to accommodate both types of trajectories. Moreover, since the duration of the increasing period is unknown, the model should be able to model both rapid and slow decays after the initial increase. Hence, the following 3 trajectories are of interest:

1. Upon infusion, the CAR‐T cell expansion increases but quickly decays to zero.
2. Upon infusion, the CAR‐T cell expansion increases and is maintained high for some time before decaying to zero.
3. Upon infusion, the CAR‐T cell expansion decreases straight to zero without increasing.

Furthermore, since the aim is to identify a recommended dose level for all patients, patient‐specific trajectories are not of primary interest.

There is no clear definition for the activity of CAR‐T cells; both the magnitude and the duration of expansions have been seen as a good indicator of the biological activity. Hence, after discussions with clinicians, the following three criteria for activity will be considered:

1. The CAR‐T cells expansion at a specific time point, i.e., only the height of the expansion is relevant, the duration of expansion is irrelevant. The underlying assumption is that if the expansion does not reach certain level at the chosen time point, the treatment would be ineffective.
2. The duration with positive CAR‐T cell expansion, i.e., the height of the expansion is irrelevant; only care about the duration. The underlying assumption is that the expansion duration should be long enough for the treatment to be effective, regardless of the magnitude of the expansion.
3. The area under the CAR‐T cells expansion trajectory, i.e., both the height and duration are relevant. This criterion assume that the expansion should be both large and long enough for the treatment to be effective.

### 3.2 Efficacy Model

**FIGURE 2:**
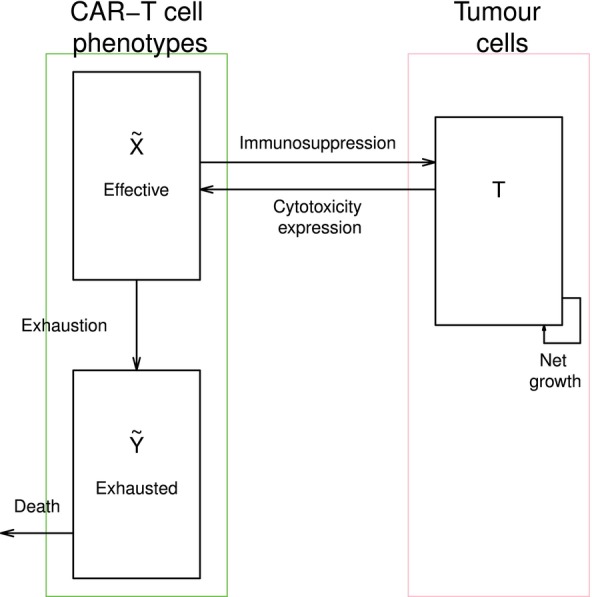
CAR‐T cells dynamics, adapted from Paixao et al. [24].

The proposed efficacy model is motivated by both the clinical considerations and the mechanistic CAR‐T kinetics. For the former, the model should be flexible enough to accommodate the above‐listed 3 types of trajectories that are clinically plausible. The functional form of the proposed efficacy model is motivated by the latter. There is a growing literature on mechanistic CAR‐T kinetic models [24, 25], which generally use a system of differential equations to describe the growth/inhibition of CAR‐T cells. Figure 2 shows a simplified version of the CAR‐T cell dynamics, in which the CAR‐T and tumour cells are shown in green and pink, respectively. The infused CAR‐T cells are either effective, i.e., interact with cancer cells and can multiply, or exhaustive, i.e., can neither interact with cancer cells nor expand. Due to the limited sample sizes in Phase I clinical trials, the estimation of the mechanistic CAR‐T kinetic models, often with more than 15 parameters, is challenging. Nevertheless, it can be shown that the expansion and decay phases of the CAR‐T cells over time can each be approximated by an exponential function (detailed in Section S1), and thus the following bi‐exponential model is proposed.

Let $Z_{i,k}$ be the CAR‐T cell expansion for patient i, i=1,…,n, at the kth measurement time $t_{k}\geq 0$, k=1,…,K, the proposed bi‐exponential model takes the form

(1) $$Z_{i,k}=d_{\left[i\right]}\left\{\exp\left(b_{1}d_{\left[i\right]}t_{k}+\log2\right)-\exp\left(b_{2}d_{\left[i\right]}t_{k}\right)\right\}+\varepsilon_{i,k},$$

for $b_{2}\leq b_{1}<0$, where $\varepsilon_{i}={\left(\varepsilon_{i,1},\ldots ,\varepsilon_{K}\right)}^{T}∼N\left(0,\sum \right)$. The first measurement is taken at the time of infusion, and thus $t_{1}=0$. It is assumed that the planned measurements times are discrete and common to all patients. The condition $b_{1}\geq b_{2}$ ensures that the mean number of cells are non‐negative. The negativity of both model parameters excludes infinite CAR‐T cells expansion. At the time of infusion, the mean CAR‐T cell expansions should equal to the dose level being infused. The factor log2 ensures $Z_{i,1}=d_{\left[i\right]}$ for all i=1,…,n. Assume all measurements are available to begin with, the problem of missing data will be discussed in Section 4.6.1.

Assuming a normal distribution can in principle lead to negative CAR‐T cell expansions, but given the magnitude of $10^{8}$ cells per litre, the model rarely fits negative values in practise. In the simulation study in Section 4, the proportions of negative cell counts are all below 1.3% among all scenarios (more details in Section S6). Other distributions defined on non‐negative integers, such as Binomial and Poisson, can also be well‐approximated by normal distributions on large magnitudes. The impact of assuming normality has been assessed in the Simulation studies. Finally, since patient‐specific trajectories are not of interest, individual random effects have not been included into the model, i.e., the serial correlation between repeated measurements in a patient has been captured only through the covariance matrix ∑.

**FIGURE 3:**
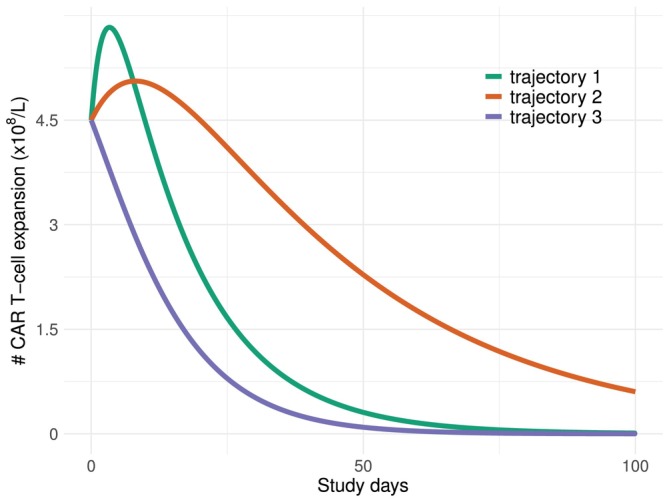
Mean CAR‐T expansions over time under dose level $d=25\times 10^{6}$ cells per kilogramme of bodyweight. Trajectory 1: $b_{1}=-1.5\times 10^{-4},b_{2}=-8\times 10^{-4}$; Trajectory 2: $b_{1}=-0.6\times 10^{-4},b_{2}=-2\times 10^{-4}$; Trajectory 3: $b_{1}=-2\times 10^{-4},b_{2}=-3\times 10^{-4}$.

Figure 3 plots the mean CAR‐T cells expansion trajectories under model (1) with 3 sets of parameter values. It shows that under different values of the model parameters $b_{1},b_{2}$, the three types of trajectories in Section 3.1 can be accommodated by the bi‐exponential model.

### 3.3 Toxicity Model

The continual reassessment method (CRM) [26] is used to model the dose‐toxicity relationship, which assumes a power model between the dose level $d_{j}$ and the DLT probability $p_{j}=\psi \left(d_{j},a\right)$, where a∈ℝ is a one‐dimensional model parameter,

(2) $$p_{j}=\psi \left(d_{j},a\right)=\alpha_{j}^{\exp\left(a\right)},\;j=1,\ldots ,J,$$

$\alpha_{j}$, j=1,…,J, are toxicity skeletons that could be interpreted as prior estimates of the DLT probability at $d_{j}$. A dose is over‐toxic if its toxicity is above ν∈[0,1]. The set of safe doses is defined as

(3) $$A≔\left\{d_{j};\mathbb{P}\left(p_{j}\geq \nu \right)<c_{\text{overdose}},j=1\ldots ,J\right\},$$

where $c_{\text{overdose}}$ is the threshold that controls overdosing.

### 3.4 Proposed Design

The toxicity and efficacy models are combined to complete the design of early phase clinical trials for CAR‐T cells. Given the data, the toxicity and efficacy models are assumed independent. This is because the main focus of this manuscript is on point estimates of the toxicity probabilities and CAR‐T cell expansions. It has been shown in Cunanan and Koopmeiners [27] that including the correlation makes little difference on point estimates, even under strong correlation. If such a correlation parameter has been included, its estimates would be close to zero. Section 4.6.2 provides a sensitivity analysis towards correlated toxicity and efficacy data.

Upon enrolling the ith cohort of patients, the toxicity model is used to select the set of safe doses, A. Then, among the safe doses, the dose level recommend to the (i+1)th patient would be the one that maximises one of the following three criteria (C1)−(C3) based on the three activity criteria in Section 3.1.

(*C*1) the CAR‐T cells expansion at time $t=T_{\text{threshold}}$,

(4) $$C_{j}^{\left(1\right)}=E_{b_{1},b_{2}}\left[d_{j}\left\{\exp\left(b_{1}d_{j}T_{\text{threshold}}+\log2\right)-\exp\left(b_{2}d_{j}T_{\text{threshold}}\right)\right\}\right],\;j=1,\ldots ,J.$$

(*C*2) the duration of CAR‐T cells expansion above a threshold $Z_{\text{threshold}}$ for $t\in \left[0,T_{\max}\right]$,

(5) $$C_{j}^{\left(2\right)}=E_{b_{1},b_{2}}\left[\sum_{t=0}^{T_{\max}}1_{\left\{d_{j}\left[\exp\left(b_{1}d_{j}t+\log2\right)-\exp\left(b_{2}d_{j}t\right)\right]\geq Z_{\text{threshold}}\right\}}\right],\;j=1\ldots ,J.$$

(*C*3) the mean area under the CAR‐T cells expansion curve (AUC) for $t\in \left[0,T_{\max}\right]$, for some longest time of interest $T_{\max}$. $d_{\left[i+1\right]}=d_{j^{*}}$ where,

(6) $$C_{j}^{\left(3\right)}=E_{b_{1},b_{2}}\left[\frac{\exp\left(b_{1}d_{j}T_{\max}\right)-2}{b_{1}}-\frac{\exp\left(b_{2}d_{j}T_{\max}\right)-1}{b_{2}}\right],\;j=1\ldots ,J.$$

Furthermore, two dose levels are “equivalent” if the metrics to be maximised are within some non‐inferiority margin. For some $c_{m}\in \left[0,1\right]$, m=1,2,3, $d_{j_{1}}$ and $d_{j_{2}}$ are equivalent under criteria $\left(C_{m}\right)$ if $C_{j_{1}}^{\left(m\right)}\in \left[\left(1-c_{m}\right)C_{j_{2}}^{\left(m\right)},\left(1+c_{m}\right)C_{j_{2}}^{\left(m\right)}\right]$. Let $d_{j^{*}}\in A$ be the dose level maximising $C_{j}^{\left(m\right)}$, j=1,…,J, the (i+1)th cohort of patient will be assigned to the lowest dose level in the safe set A among all doses equivalent to $d_{j^{*}}$. After all patients are evaluated, the final selected level will be the optimal dose. This is summarised in Algorithm 1.

ALGORITHM 1 **Early phase design for CAR T‐cells.**

1: The first cohort of patients is assigned to dose $d_{1}$.
 2: safe = True.
 3: **while** safe and i≤n **do**
 4:   **procedure** safety evaluation
 5:    Update the toxicity probabilities ${\hat{p}}_{j}=\psi \left(d_{j},\tilde{a}\right)$ according to toxicity model (2), where $\tilde{a}$ is the current estimate of a given toxicity data, j=1,…,J.
 6:    Find the set of safe doses A according to Equation (3)
 7:    **if** A=ϕ **then**
 8:      safe = False 
 9:    **end if**
10:   **end procedure**
11:   **procedure** efficacy evaluation
12:     Fit the efficacy model (1). Obtain the current estimates of $b_{1},b_{2}$ as ${\hat{b}}_{1},{\hat{b}}_{2}$.
13:     $d_{j^{*}}=\arg\max_{d_{j}\in A}C_{j}^{\left(m\right)}$, m=1,2,3, according to Equations (4)‐(6).
14:     $d_{\left[i+1\right]}=\min\left\{d_{j}\in A:C_{j}^{\left(m\right)}\in \left[\left(1-c_{m}\right)C_{j^{*}}^{\left(m\right)},\left(1+c_{m}\right)C_{j^{*}}^{\left(m\right)}\right]\right\}$.
15:   **end procedure**. 
16:   Assign the (i+1)th cohort to dose $d_{\left[i+1\right]}$.
17:   i←i+1.
18: **end while**
19: **if** safe **then**
20:   The final recommended dose level is $d_{\left[n+1\right]}$
21: **else**
22:   No recommended dose
23: **end if**

### 3.5 Model Estimation

The model parameters are estimated under the Bayesian framework. For the toxicity model (2), the model parameter a is assumed to follow a normal prior distribution $N\left(0,\sigma_{T}^{2}\right)$, where $\sigma_{T}^{2}$ is the prior variance. Let $Y_{i}$, i=1,…,n, be the binary DLT outcome of the ith patient, where $Y_{i}=1$ if the ith patient has an DLT, $Y_{i}=0$ otherwise. Let $p_{\left[i\right]}≔\psi \left(d_{\left[i\right]},a\right)$, then, $Y_{i}$ follows a Bernoulli distribution $Y_{i}∼\mathrm{Bin}\left(1,p_{\left[i\right]}\right)$. After evaluating the ith patient, the posterior distribution of the model parameter a can be calculated, and the posterior mean $\tilde{a}$ is plugged into Equation (2) and (3) to update the estimates of DLT probabilities and the set of safe doses.

For the efficacy model in Equation (1), to minimise the number of model parameters, so that they can be estimated based on the small sample size available, the following autoregressive structure of the covariance matrix ∑ is assumed.

(7) $$\sum =\sigma_{E}^{2}\left(\begin{array}{llllll} 1 & \rho & \rho^{2} & \cdots & \rho^{K-1} & \\ \rho & 1 & \rho & \cdots & \rho^{K-2} & \\ \rho^{2} & \rho & 1 & \cdots & \rho^{K-3} & \\ \vdots & \vdots & \vdots & \ddots & \vdots & \\ \rho^{K-1} & \rho^{K-2} & \rho^{K-3} & \cdots & 1 & \end{array}\right),$$

for some ρ∈[0,1]. Under the Bayesian framework, the prior distribution on ${\left(b_{1},b_{2},\sigma_{E}^{2},\rho \right)}^{T}$ are as follows.

$$\begin{array}{cc} b_{2}\;|\;\left({b_{1},\sigma }_{E}^{2}\right) & ∼\mathrm{TN}\left(\mu_{2},\sigma_{E}^{2}s_{2}^{2}\right)\\ \;b_{1}\;|\;\sigma_{E}^{2} & ∼N\left(\mu_{1},\sigma_{E}^{2}s_{1}^{2}\right)\\ \sigma_{E}^{2} & ∼\mathrm{IG}\left(\gamma ,\beta \right)\\ \rho & ∼U\left(0,1\right), \end{array}$$

with hyperparameters $\left(\mu_{1},\mu_{2},s_{1}^{2},s_{2}^{2},\gamma ,\beta \right)$, where TN is the truncated normal distribution with $b_{2}\leq b_{1}<0$, ℐG(γ,β) refers to the inverse Gamma distribution with shape and scale parameters γ,β, respectively. The posterior distribution can be obtained via a MCMC algorithm using rjags.

## 4 Evaluations of Proposed Design

### 4.1 Setting

The operational characteristics of the design are evaluated through extensive simulation studies. The following setting has been considered. Suppose there are 4 dose levels, $25,75,225,450\times 10^{6}$ CAR‐T cells per kilogramme of bodyweight. A dose is considered safe if the DLT risk is less than 30%, i.e., ν=0.3. The toxicity skeleton $\alpha_{j}$, j=1,…,J in model 2 are selected using the getprior() function from the dfcrm R package [28], which generally leads to good model performance under the CRM [29]. The skeleton obtained is $\left(\alpha_{j};j=1,\ldots ,4\right)=\left(0.11,0.20,0.31,0.42\right)$.

Discussions with biologists suggest that the CAR‐T cells expansion after 2 weeks of infusion seems to be important, and thus under efficacy criterion (C1) in Equation (4), the threshold is set to $T_{\text{threshold}}=14$ days. In criterion (C2), $Z_{\text{threshold}}=25$ ($\times 10^{6}$ CAR‐T cells per kilogramme of bodyweight) in Equation (5) so that at the lowest dose level, if the infused CAR‐T cells do not expand, its expansion duration will be zero. Under criterion (C3), the study period in real trials is typically 3–4 months after infusion, and thus $T_{\max}=91$ days in Equation (6), 13 weeks after infusion. In the definition of equivalent dose levels, it is suggested that a 10% difference in $C_{j}^{\left(m\right)}$, m=1,2,3, could be considered negligible, and thus we set $c_{1}=c_{2}=c_{3}=0.1$. Sensitivity analysis on the equivalence margins are detailed in Section S8, where 5% and 15% margins are assessed.

The sample size is assumed to be n=10, and patients are enrolled as 5 cohorts of 2. Additional results for n=20 are shown in Section S5. The starting dose is set to the lowest dose $d_{1}$, and additional results when starting at $d_{2}$ are shown in Table S7. We start by assuming the measurements are taken once per week for 14 weeks. The impact of irregular measurements will be assessed at the end of this section. The objective is to find the optimal dose that has the highest efficacy within the set of safe doses.

### 4.2 Non‐Parametric Benchmark for CAR‐T Cells

Non‐parametric benchmarks provide upper bounds on the proportion of correct selection (PCS) of the OD under given scenarios, regardless of the dose‐finding design, which was developed by O'Quigley et al. [20] for a single binary toxicity endpoint, and extended to designs with a binary toxicity endpoint and a continuous efficacy endpoint in Mozgunov et al. [22]. Theoretical guarantee for the benchmarks to provide upper bounds on the PCS comes from the use of “complete information”, i.e., measurements on all dose levels. Whereas, in reality, the observed data only provides “partial information”, which are measurements only on the dose level the patients received. Below, we construct a non‐parametric benchmark specifically for CAR‐T cells, which follows the same rationale as the previous works.

For toxicity model, for any patient i, only *partial information* can be obtained. That means, if a DLT is observed, the dose $d_{\left[i\right]}$ and all levels above are toxic for patient i, while nothing can be said about doses below $d_{\left[i\right]}$, and similarly if a non‐DLT is observed. On the other hand, the non‐parametric benchmark assumes *full information*, which means the toxicity result at all doses can be obtained for all patients. For efficacy model, partial information means measurements of CAR‐T expansion can only be obtained at dose $d_{\left[i\right]}$ and no other doses, while complete information assumes measurements at all levels for all patients.

Hence, let $u_{i}^{T}∼U\left(0,1\right)$ be the toxicity profile of patient i, and let $Y_{i,j}$ be the toxicity outcome of patient i at dose $d_{j}$. Then, enrolling patient i to any dose with DLT probability larger than $u_{i}$ will results in a DLT, while enrolling to any dose with DLT probability smaller than $u_{i}$ gives a non‐DLT, i.e., $Y_{i,j}=\;1\left\{R_{j}>u_{i}^{T}\right\}$, where $R_{j}$ is the true DLT probability of $d_{j}$. The DLT probability at $d_{j}$ can therefore be estimated as ${\hat{p}}_{j}={\bar{Y}}_{j}$ for $Y_{j}=\left(Y_{i,j};i=1,\ldots ,n\right)$. To define the set of safe doses A, the probability $\mathbb{P}\left(p_{j}\geq \nu \right)$ can be approximated by $1-\Phi \left(\frac{\sqrt{n}\left(\nu -{\hat{p}}_{j}\right)}{\sqrt{{\hat{p}}_{j}\left(1-{\hat{p}}_{j}\right)}}\right)$, where Φ(⋅) refers to the cumulative distribution function (c.d.f.) of N(0,1).

Among the safe doses $d_{j}\in A$, let $Z_{i,j,k}$ be the CAR expansion of patient i when receiving dose $d_{j}$ at its kth measurement time $t_{k}$, then $Z_{i,j,k}=\mu_{j,k}+\varepsilon_{i,k}$, where $\mu_{j,k}$ is the true CAR‐T expansions under the given efficacy scenario for dose $d_{j}$ at time $t_{k}$, $\varepsilon_{i}∼N\left(0,\sum_{x}\right)$ for $\sum_{x}=\frac{1}{\sigma_{E}^{2}}\sum$ and ∑ defined in Equation (7). Let $F_{j,k}$ be the c.d.f. of $Z_{i,j,k}$, and let $F_{j,k}^{-1}$ be the inverse c.d.f, by the probability integral transform, $F_{j,k}^{-1}\left(U\right)∼F_{j,k}$ for U∼U(0,1), the standard uniform distribution. Let $u_{i,k}^{E}∼U\left(0,1\right)$ be the efficacy profile of patient i at time $t_{k}$, then $Z_{i,j,k}=F_{j,k}^{-1}\left(u_{i,k}^{E}\right)$ for all j=1,…,J. The profiles satisfy $u_{i,k}^{E}=\Phi \left(x_{i,k}\right)$ and $x_{i}=\left(x_{i,k};k=1,\ldots ,K\right)∼N_{K}\left(0,\sum \right)$, where the covariance ∑ is as defined in Equation (7). The escalation criteria (C1)−(C3) are defined in the same manner as in Section 3.4. This is summarised in Algorithm 2 below.

ALGORITHM 2 **Non‐parametric benchmark for CAR T‐cells.**

1: **procedure** toxicity model
 2:   Simulate toxicity profile $u_{i}^{T}∼U\left(0,1\right)$ independently for i=1,…,n.
 3:   Let $Y_{i,j}=1$ if $R_{j}>u_{i}^{T}$, =0, otherwise, for j=1,…,J,i=1,…,n. Store $Y_{j}=\left(Y_{1,j},\ldots ,Y_{n,j}\right)$.
 4:   Estimate the DLT probability at $d_{j}$, ${\hat{p}}_{j}={\bar{Y}}_{j}$.
 5:   Estimate the probability of overdosing $P\left(p_{j}\geq \nu \right)=1-\Phi \left(\frac{\sqrt{n}\left(\nu -{\hat{p}}_{j}\right)}{\sqrt{{\hat{p}}_{j}\left(1-{\hat{p}}_{j}\right)}}\right)$. Construct the set of safe doses $A=\left\{d_{j}:P\left(p_{j}\geq \nu \right)<c_{\text{overdose}}\right\}$.
 6: **end procedure**
 7: **procedure** efficacy model
 8:   Simulate $x_{i}∼N_{K}\left(\mu ,\sum_{x}\right)$, where $\mu ={\left(0,\cdots ,0\right)}^{T}$, and $\sum_{x}=\left(\begin{array}{lllll} 1 & \rho & \rho^{2} & \rho^{3} & \cdots \\ \rho & 1 & \rho & \rho^{2} & \cdots \\ \rho^{2} & \rho & 1 & \rho & \cdots \\ \vdots & \vdots & \vdots & \ddots & \vdots \end{array}\right)$, i=1,…,n.
 9:   Obtain efficacy profiles $u_{i,k}=\Phi \left(x_{i,k}\right)$, k=1,…,K, i=1,…,n.
10:   Transform profiles to CAR‐T expansions $Z_{i,j,k}=F_{j,k}^{-1}\left(u_{i,k}\right)$ for i=1,…,n, j=1,…,J, k=1,…,K. Store $Z_{j,k}=\left(Z_{1,j,k},\ldots ,Z_{n,j,k}\right)$.
11:   **for** m in 1, 2, 3 **do**
12:     Calculate $C_{j}^{\left(m\right)}$ according to Equation (4)‐(6) based on $Z_{j}=\left(Z_{i,j,k}:i=1,\ldots ,n,k=1,\ldots ,K\right)$.
13:     $d_{j^{*}}=\arg\max_{d_{j}\in A}C_{j}^{\left(m\right)}$.
14:     $j_{m}^{*}=\min\left\{d_{j}\in A:C_{j}^{\left(m\right)}\in \left[\left(1-c_{m}\right)C_{j^{*}}^{\left(m\right)},\left(1+c_{m}\right)C_{j^{*}}^{\left(m\right)}\right]\right\}$
15:   **end for**
16: **end procedure**
17: Repeat steps 1‐ 16 for simulated trials s=1,…,S Use $N_{c}^{\left(j\right)}=\frac{1}{S}\sum_{s=1}^{S}1\left\{j_{c}^{*}=j\right\}$, c=1,2,3, as the proportion of selecting $d_{j}$ under criterion (C1),(C2),(C3)

### 4.3 Scenarios

The operational characteristics will be assessed on

1. Accuracy: the proportion of correct selection (PCS) of the optimal dose;
2. Safety: the proportion of patients receiving unsafe doses with DLT probability higher than 30%.

Based on real trials on CAR‐T cells, very few DLTs have been reported. Hence, we consider two scenarios of DLT probabilities:

T1:(0.01,0.05,0.10,0.15);T2:(0.05,0.10,0.20,0.35),

where each bracket gives the DLT probabilities of $d_{1},\ldots ,d_{4}$. In scenario T1, all doses have the probability of DLT lower than 30% and are all safe. In scenario T2, the highest level $d_{4}$ is not safe and the OD should be chosen from the first three doses.

For efficacy scenarios, we consider the following 5 data generating mechanisms, where the model is misspecified under scenarios E2–E5.

Scenario E1 generates the CAR‐T expansion from the proposed bi‐exponential model (1) with true model parameter values $b_{1}=-1.1e-4$, $b_{2}=-8e-3$, $\sigma_{E}=1\times 10^{8}$, and ρ=0.5.

Under scenario E2, a random effect $u_{i}$ for patient i has been added to induce the serial correlation between repeated measurements. Hence, the CAR‐T expansion for patient i at time $t_{k}$ is simulated according to the following conditional non‐linear mixed effect model

$$Z_{i,k}=d_{\left[i\right]}\left\{\exp\left(b_{1}d_{\left[i\right]}t_{k}+\log2\right)-\exp\left(b_{2}d_{\left[i\right]}t_{k}\right)\right\}+u_{i}+\varepsilon_{i,k},$$

i=1,…,n, k=1,…,K, where $u_{i}$ is a realisation of $U∼N\left(0,\omega^{2}\right)$, $u_{i}⟂\varepsilon_{i}$, i=1,…,n, and all other notations are as defined in Equation (1). The same values of $b_{1}=-1.1e-4,b_{2}=-8e-3$ are used, and we fix $\omega =1\times 10^{8}$ cells per litre. The marginal model in Equation (1) will be fitted on this data, and the impact of ignoring the random effect $u_{i}$ will be assessed.

Scenario E3 models the case of two subpopulations among the patients. The infused cells would either exhaust and progressively get eliminated from the blood (called the “no expansion class”) or proliferate and be maintained over some duration before being eliminated (called the “positive expansion class”). We simulate CAR‐T expansion data according to this two‐subpopulation conjecture. Let $G_{i}$ be the subpopulation patient i belongs to, $G_{i}=0$ corresponds to the no expansion class, and $G_{i}=1$ the positive expansion class. It is believed that the probability of belonging to the positive expansion class may increase with dose level, and thus we simulate $G_{i}∼ℬ\text{in}\left(1,0.2p_{\left[i\right]}\right)$, and $p_{\left[i\right]}=0.2,0.4,0.6,0.8$ for patients assigned to doses $d_{1},\ldots ,d_{4}$, respectively. Given $G_{i}$, the CAR‐T expansion is simulated from

$$Z_{i,t}=d_{\left[i\right]}\left\{\exp\left(b_{1}d_{\left[i\right]}+G_{i}\log2\right)-G_{i}\exp\left(b_{2}d_{\left[i\right]}t\right)\right\}+\varepsilon_{i,t}.$$

The true values of model parameters $b_{1},b_{2}$ are the same as scenario E1 and E2, $b_{1}=-1.1e-4,b_{2}=-8e-3$. Model (1) with only the positive expansion class will be fitted to this data generated with two sub‐populations, and the impact of ignoring the no expansion class will be assessed.

Scenario E4 is based on the observed CAR‐T expansion data reported in Cwynarski et al. [23] (if the data is not available at a certain time $t_{k}$, it is calculated by linear interpolation). Given the observed CAR‐T expansion $z_{i,k}$ for patient i at time $t_{k}$, the CAR‐T expansion data is simulated according to $Z_{i,k}=z_{i,k}+\varepsilon_{i,k}$, where $\varepsilon_{i}∼N\left(0,\sum \right)$, ∑ is as defined in Equation (7) with $\sigma_{E}=1\times 10^{8}$ cells per litre and ρ=0.5.

Scenario E5 is designed to assess the impact of assuming a normal distribution for CAR‐T cell expansions. Instead, the CAR‐T cells expansion $Z_{i,k}$ is generated from a negative binomial distribution with mean $\mu_{i,k}=d_{\left[i\right]}\left\{\exp\left(b_{1}d_{\left[i\right]}t_{k}+\log2\right)-\exp\left(b_{2}d_{\left[i\right]}t_{k}\right)\right\}+u_{i}$ and dispersion parameter $\sigma_{E}^{2}$. The random effect $u_{i}$, same as defined in scenario E2, is used to account for serial correlations between repeated measurements. The parameter values are the same as those used in scenarios E1‐E3, $b_{1}=-1.1e-4$, $b_{2}=-8e-3$, and $\omega =1\times 10^{8}$.

**FIGURE 4:**
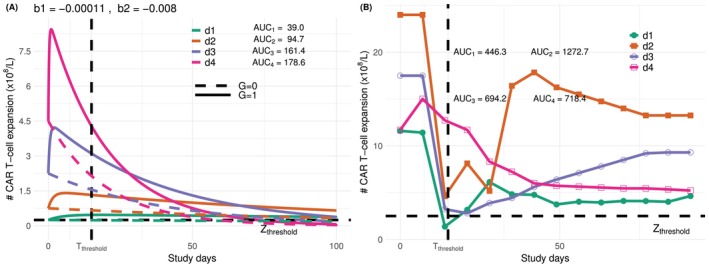
(A) G=1 shows the mean CAR‐T expansion trajectories under scenario E1, E2, E5, and the positive expansion class in E3, G=0 shows the no expansion class in E3. (B) The observed CAR‐T expansion from Cwynarski et al. [23]. $T_{\text{threshold}}=14$ days, $Z_{\text{threshold}}=25\times 10^{6}$ cells per kilogramme of bodyweight.

The five efficacy scenarios E1—E5 are presented in Figure 4. The solid lines in (Panel A) plot the mean CAR‐T expansion trajectories under scenario E1, E2, E5, and the G=1 class in E3. The dashed lines in (Panel A) plots the G=0 class in scenario E3. (Panel B) plots the observed CAR expansion data.

Under scenarios E1, E2, and E5, since the true values of model parameters are known, true values of the escalation criteria $C_{j}^{\left(m\right)}$, j=1,…,4, m=1,2,3, can be calculated (see Table 1). Under scenario E3, the mean values of $C^{\left(m\right)}$ between the two sub‐populations are calculated via simulations. For r=1,…,R, simulate $G_{i}^{\left(r\right)}∼ℬ\text{in}\left(1,0.2p_{\left[i\right]}\right)$ and calculate $C_{j,r}^{\left(m\right)}$ for j=1,…,4. When $G_{i}^{\left(r\right)}=0$, the AUC under the no expansion class is $\left(e^{b_{1}d_{j}T_{\max}}-1\right)/b_{1}$ for j=1,…,4. Then, the mean values are calculated as $C_{j}^{\left(m\right)}=\frac{1}{R}\sum_{r}C_{j,r}^{\left(m\right)}$, where we took $R=5\times 10^{4}$, shown in the row labelled E3 in Table 1. Under scenario E4, there is no true model since it is based on observed data. Hence, true values of $C_{j}^{\left(m\right)}$ are estimated from the observed values. Based on linear interpolation, $C_{j}^{\left(1\right)}$ are the observed CAR‐T cells expansions on day 14. $C_{j}^{\left(2\right)}$ are the number of days the observed trajectories stay above $0.25\times 10^{8}$, and $C_{j}^{\left(3\right)}$ are the area under the observed trajectories, estimated using the Riemann sum. The values are shown in the last row of Table 1.

**TABLE 1:** True values of the escalation criteria $C_{j}^{\left(m\right)}$ for j=1,…,4 and m=1,2,3. ODs highlighted in bold.

	Dose levels				Dose levels				Dose levels			
	$d_{1}$	$d_{2}$	$d_{3}$	$d_{4}$	$d_{1}$	$d_{2}$	$d_{3}$	$d_{4}$	$d_{1}$	$d_{2}$	$d_{3}$	$d_{4}$
Scenario	$C^{\left(1\right)}\left(\times 10^{8}\right)$				$C^{\left(2\right)}$ (days)				$C^{\left(3\right)}\left(\times 10^{8}\right)$			
T1‐E1	0.5	1.3	3.1	**4.3**	**91**	91	91	72	39.0	94.7	**161.4**	178.6
T1‐E2	0.5	1.3	3.1	**4.3**	**91**	91	91	72	39.0	94.7	**161.4**	178.6
T1‐E3	0.3	0.9	2.5	**3.9**	18.5	**91**	89.8	69.2	23.8	66.9	129.5	**161.1**
T1‐E4	1.6	5.1	3.2	**12.6**	**87**	91	91	91	446.3	**1272.7**	694.2	718.4
T1‐E5	0.5	1.3	3.1	**4.3**	**91**	91	91	72	39.0	94.7	**161.4**	178.6
T2‐E1	0.5	1.3	**3.1**	4.3	**91**	91	91	72	39.0	94.7	**161.4**	178.6
T2‐E2	0.5	1.3	**3.1**	4.3	**91**	91	91	72	39.0	94.7	**161.4**	178.6
T2‐E3	0.3	0.9	**2.5**	3.9	18.5	**91**	89.8	69.2	23.8	66.9	**129.5**	161.1
T2‐E4	1.6	**5.1**	3.2	12.6	**87**	91	91	91	446.3	**1272.7**	694.2	718.4
T2‐E5	0.5	1.3	**3.1**	4.3	**91**	91	91	72	39.0	94.7	**161.4**	178.6

In simulation studies, we combine the two toxicity scenarios T1, T2 with the five efficacy scenarios E1, …, E5, to obtain 10 toxicity‐efficacy scenarios. The OD is the level that optimises the activity criterion among the safe doses. For example, under scenario T2‐E1 and under criterion (C1), only the first 3 doses are safe among which, the one that maximises the CAR‐T cells expansion at 14 days is $d_{3}$ and no other dose is equivalent to $d_{3}$. Thus, $d_{3}$ is the OD. The ODs under the 10 combined toxicity‐efficacy scenarios are highlighted in **bold** in Table 1.

### 4.4 Choice of Design Parameters

The design shown in Algorithm 1 requires a number of design parameters that need to be fixed before starting the trial. These parameters can be chosen either based on guidance from clinicians or based on statistical considerations, i.e., choose the set of values that optimise the design performance across various scenarios. The values chosen under the latter approach is called *operational priors*. We suggest a way to calibrate parameters based on statistical considerations and refer readers to Mozgunov et al. [30] for working with clinicians to select design parameters.

The toxicity model requires to fix two parameters, the prior variance $\sigma_{T}^{2}$ and the threshold for overdose $c_{\text{overdose}}$. The efficacy design requires 6 design parameters, the prior mean and variance for $\left(b_{1},b_{2}\right)$, $\mu_{1},\mu_{2},s_{1}^{2},s_{2}^{2}$, and the hyperparameters for the inverse gamma distribution, γ,β. By convention, setting $\sigma_{T}^{2}=1.34$ would lead to reasonable design performance [31]. One way of calibrating the parameters is to conduct a “cyclic search” [32]. Explicitly, a grid of potential values for each design parameter is specified, fixing the values of one but one parameter, we search over the grid of the remaining parameter space for the optimal value that maximises the mean PCS. The algorithm iterates across all parameters until it stays at the same set of values over two cycles, where one cycle is defined as searching over each design parameter once. The grids of the 7 parameters were chosen as

- The threshold for overdose control $c_{\text{overdose}}\in \left\{0.15,0.20,0.25,0.30\right\}$;
- The prior means $\mu_{1},\mu_{2}\in \left\{-2,-1,0,1,2\right\}\times 10^{-4}$.
- The prior variances $s_{1}^{2},s_{2}^{2}\in \left\{1,2,5\right\}\times 10^{-7}$;
- The shape parameter of inverse gamma γ∈{1,2,5};
- The scale parameter of inverse gamma β∈{1,2,5,10}.

The calibration is based on the easiest and hardest toxicity‐efficacy scenarios among the ten specified in Section 4.3, where scenario difficulty is measured by the PCS obtained with the non‐parametric benchmark proposed in Algorithm 2. Under the efficacy criterion (C1), the easiest (best PCS) and hardest (worst PCS) scenarios are T1‐E1 and T2‐E2 with upper bounds on the PCS 98.82% and 20.02%, respectively. Under efficacy criterion (C2), the easiest and hardest scenarios are T1‐E4 and T1‐E1 with upper bounds on PCS 99.84% and 48.92%, respectively. Under criterion (C3), the easiest and hardest scenarios are T1‐E4 and T2‐E3 with upper bounds on PCS 99.88% and 27.24%, respectively. We choose the design parameters that maximise the mean PCS under these two scenarios, the resulted operational prior under criterion (C1) is $\left(c_{\text{overdose}},\mu_{1},\mu_{2},s_{1}^{2},s_{2}^{2},\gamma ,\beta \right)=\left(0.30,-1\times 10^{-4},-1\times 10^{-4},1\times 10^{-7},5\times 10^{-7},2,5\right)$, under (C2) is $\left(0.25,1\times 10^{-4},0,2\times 10^{-7},2\times 10^{-7},2,10\right)$, under (C3) is $\left(0.30,-1\times 10^{-4},-2\times 10^{-4},2\times 10^{-7},1\times 10^{-7},1,5\right)$.

On average, the algorithm takes 2 cycles to converge. Using the above grids (4, 5, 5, 3, 3, 3, 4 values for $c_{\text{overdose}}$, $\mu_{1}$, $\mu_{2}$, $s_{1}^{2}$, $s_{2}^{2}$, γ, β, respectively), each cycle requires simulating under 4+5+5+3+3+3+4=27 values of design parameters, and thus 2 cycles takes 3×27=54 values. Only the easiest and hardest scenarios are used for calibration, 500 simulations have been done for each value, and it takes around 22 s to run one simulated trial with sample size n=10 on a regular laptop. Hence, in total, the calibration process takes 54×2×500×22=1188000 seconds =13.75 days. Furthermore, under each values of design parameters, the simulations can be conducted in a parallel manner, and it is common for modern laptops to have at least 4 cores. In that case, the computing time can be reduced to roughly 13.75/4≈3.5 days. This is a very affordable computation time for practitioners to run.

### 4.5 Results

**FIGURE 5:**
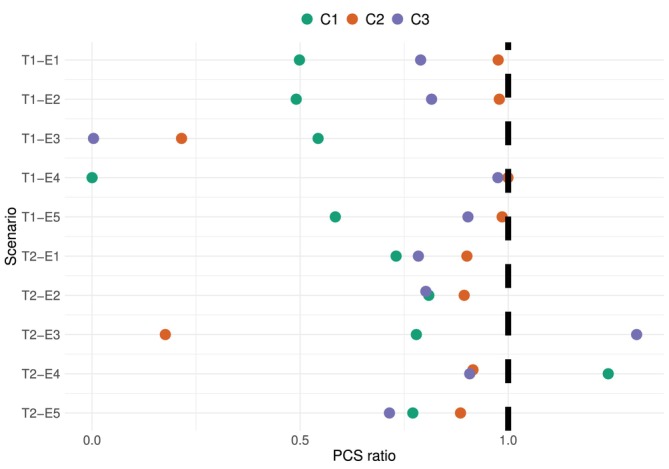
Ratio between the PCS under our proposed design and the upper bound given by the non‐parametric benchmark. Colours correspond to the three efficacy criteria. Sample size n=10, based on $10^{4}$ simulations.

Under each of the three efficacy criteria (C1)−(C3), the PCS under the ten scenarios are estimated based on $10^{4}$ simulations. Figure 5 plots the ratio between the PCS under our proposed design and the upper bound of PCS given by the non‐parametric benchmark in Algorithm 2. A ratio close to 1 corresponds to good operational characteristics. The proportions of selecting each dose under each scenario are provided in Table 2.

**TABLE 2:** The proportion of selecting each dose by the proposed design in Algorithm 1 under the 10 toxicity‐efficacy scenarios and three efficacy criteria based on $10^{4}$ simulations.

	Dose levels				Dose levels				Dose levels			
	$d_{1}$	$d_{2}$	$d_{3}$	$d_{4}$	$d_{1}$	$d_{2}$	$d_{3}$	$d_{4}$	$d_{1}$	$d_{2}$	$d_{3}$	$d_{4}$
Scenario	$C^{\left(1\right)}$				$C^{\left(2\right)}$				$C^{\left(3\right)}$			
T1‐E1	0.43	24.57	32.33	**40.52**	**97.60**	0	0	0	3.48	18.45	**75.61**	0.92
T1‐E2	1.72	24.57	31.47	**41.81**	**97.77**	0	0	0	3.87	17.96	**75.64**	1.38
T1‐E3	3.02	18.53	32.33	**45.26**	74.74	**17.78**	0	0	3.18	15.17	80.3	**0.30**
T1‐E4	3.45	95.26	0	**0**	**98.06**	0	0	0	2.33	**96.16**	0	0
T1‐E5	1.71	20.58	31.91	**44.70**	**97.44**	0	0	0	2.8	17.05	**69.79**	8.40
T2‐E1	7.33	39.22	**35.78**	10.34	**89.77**	0	0	0	10.74	28.82	**52.23**	0.36
T2‐E2	6.03	44.4	**27.59**	15.09	**88.63**	0	0	0	10.37	26.63	**54.43**	0.46
T2‐E3	7.33	40.95	**31.90**	13.36	70.82	**14.05**	0	0	10.34	23.67	**58.50**	0.03
T2‐E4	12.07	**82.76**	0	0	**88.70**	0	0	0	7.68	**84.50**	0.03	0.03
T2‐E5	5.85	44.82	**27.65**	11.94	**89.52**	0	0	0	9.26	30.33	**49.57**	3.05

*Note*: The ODs are highlighted in bold.

**FIGURE 6:**
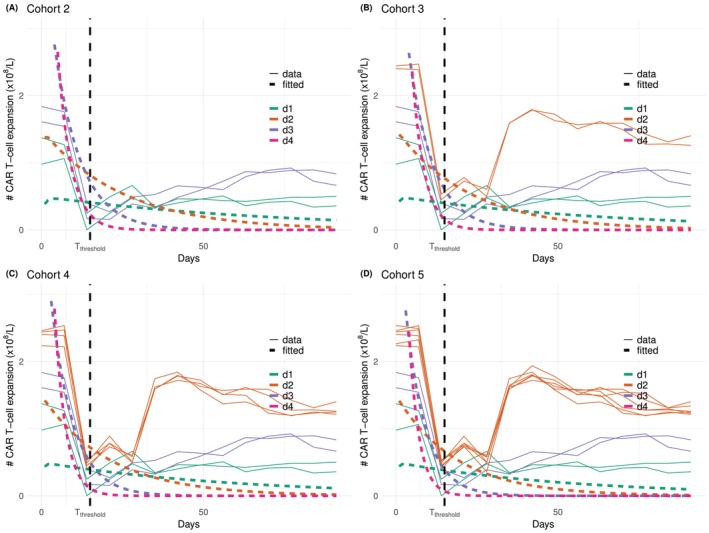
CAR‐T cells trajectories under scenario T1‐E4 and escalation criteria (C1) at cohort 2 (Panel A), 3 (Panel B), 4 (Panel C), 5 (Panel D). Simulated CAR‐T cells expansions in solid lines and fitted bi‐exponential models in dashed lines.

Under escalation criterion (C1) (shown in green), the performance of the proposed design is generally good except for scenario T1‐E4, where the OD, $d_{4}$, has not been selected and $d_{2}$ has been selected with 95% frequency, as shown in Table 2. Nevertheless, scenario E4 is designed to be particularly hard for criterion (C1). Figure 6 plots the trajectories of the simulated CAR‐T cells expansion (solid lines) and the fitted bi‐exponential models (dashed lines) upon evaluating each cohort of patients. The true CAR‐T cells trajectories under $d_{1},d_{2},d_{3}$, shown in Figure 4 (Panel B), drop to close to zero at 14 days before they increase back to $0.5\times 10^{8}$ under $d_{1}$, $1\times 10^{8}$ under $d_{3}$, and $1.5\times 10^{8}$ under $d_{2}$. The bi‐exponential distribution, however, cannot accommodate this double expansion, and thus the fitted trajectories are far from the observed data at the second expansion. Moreover, the model is misspecified at doses $d_{2}$, $d_{3}$ and $d_{4}$ in this setting, leading to biassed recommendation.

**FIGURE 7:**
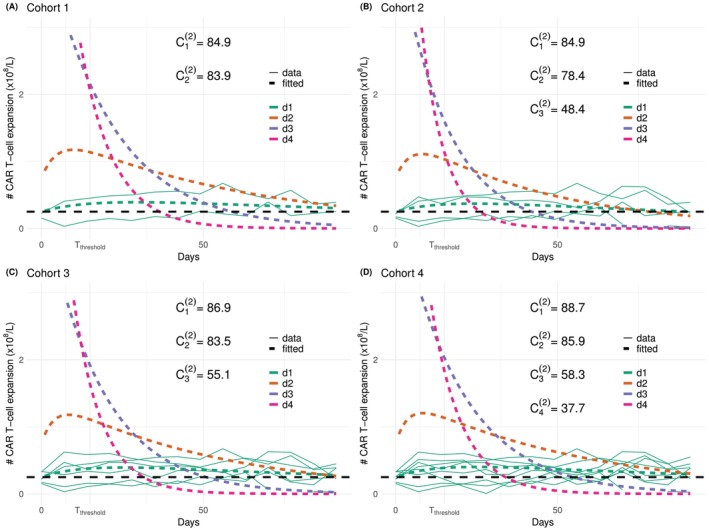
CAR‐T cells trajectories under scenario T1‐E3 and escalation criterion (C2). Simulated data in solid lines and fitted bi‐exponential models in dashed lines.

Under escalation criterion (C2) (shown in orange), the PCS ratios shown in Figure 5 are around 1 under scenarios E1, E2, E4, E5, and around 0.2 under scenario E3. This is because we chose $Z_{\text{threshold}}=d_{1}$, and thus a tiny estimation errors would result in big difference in the estimated $C_{1}^{\left(2\right)}$. Figure 7 plots the data trajectories and fitted bi‐exponential models for one simulated trial. The fitted trajectory under $d_{1}$ is in fact very close to the data, nevertheless, $C_{1}^{\left(2\right)}$ is still largely overestimated. This overestimation then leads to all patients being assigned to $d_{1}$, and thus information from the other three doses cannot help to correct this bias. Decreasing $Z_{\text{threshold}}$ from $0.25\times 10^{8}$ to $0.2\times 10^{8}$, for instance, would immediately increase the PCS from the current 18%–53%, and the PCS ratio from 0.2 to 0.6, as shown in the Supporting Informations.

**FIGURE 8:**
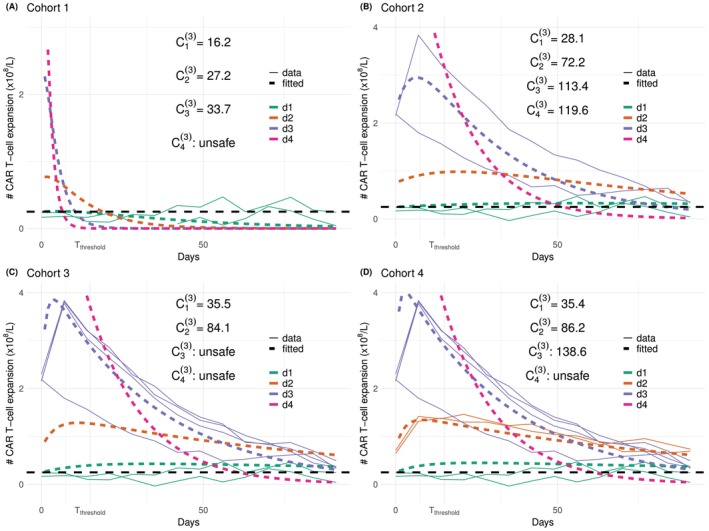
CAR‐T cells trajectories under scenario T1‐E3 and escalation criterion (C3). Simulated data in solid lines and fitted bi‐exponential models in dashed lines.

Under criterion (C3) (shown in purple), the PCS ratios are all larger than 80% except for scenario T1‐E3, where the OD $d_{4}$ has only been selected 0.3% of the time, whereas $d_{3}$ is selected 80% of the time. Note that under the toxicity scenario T2, the ratio is indeed close to 1, and thus the undermined accuracy under T1‐E3 is largely due to the toxicity probability at $d_{4}$ being over‐estimated and $d_{4}$ being considered unsafe. Figure 8 shows the cohort‐by‐cohort behaviour under this scenario. The fitted trajectories are close to the true trajectories, the AUC under $d_{3}$ can be over‐estimated slightly, but the main reason for rarely selecting $d_{4}$ is due to the sample size being too small for the toxicity model to accurately estimate the DLT probability at $d_{4}$.

### 4.6 Sensitivity Analysis

#### 4.6.1 Missing Measurements

So far, it has been assumed that all patients had no missing measurement times, which are evenly spaced once a week. In reality, the measurements are taken much more frequently at the beginning of the study period, roughly once every 2 days during the first 2 weeks after infusion, after when, patients start to have missing measurements. This section assesses the impact of having incomplete measurement times.

With the current structure of the covariance matrix ∑, as long as there exists a protocol on the days the measurements should be taken, model (1) can be fitted even if some measurements are missing. In the simulation below, we assess the following two missing mechanisms [33], Chapter 1.

- Missing completely at random (MCAR): all patients would take the first four measurements at day 0, 7, 14, and 21. Then, among the further 10 measurements, each patient will randomly take 0 to 10 of them.
- Missing at random (MAR): all patients would take the first four measurements at day 0, 7, 14, and 21. Then, patients whose CAR‐T cells expansions are larger than $1\times 10^{8}$ cells per litre at day 21 would have complete measurements afterwards, whereas the others would have no further measurement.

**FIGURE 9:**
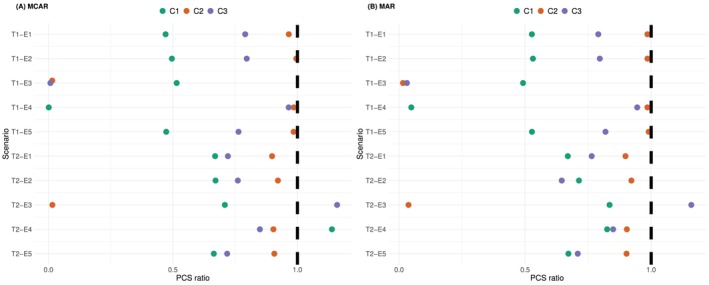
The ratio between the PCS under irregular measurement times and the one under the non‐parametric benchmark. Colours correspond to the 3 efficacy criteria. Sample size n=10, based on $10^{4}$ simulations.

The ratios of the PCS under incomplete measurement times to the non‐parametric benchmark are compared in Figure 9. The probabilities of selecting each dose in each scenario are detailed in Supporting Informations. (Panel A) considers the MCAR missing mechanism. Overall, this does not bias the estimates, the PCS ratios are very similar to that under regular measurements shown in Figure 5 in all scenarios and all escalation criteria except for scenarios T1‐E3 and T2‐E3 under (C2). Even without missing measurements, the PCS under these two scenarios were low due to the fact that $Z_{\text{threshold}}=d_{1}$. With missing measurements, the noise would have an even larger impact on the estimation, and thus the PCS are even lower, as expected.

The MAR missing mechanism considered in (Panel B) shows a very similar performance to (Panel A). Having the missing mechanism depending on the CAR‐T cells expansion does not bring further bias to the proposed design. The biggest difference between the PCS under MAR and complete measurements is scenario T2‐E4 under (C1). The proportion of selecting the MTD $d_{2}$ decreases from approximately 80%–50%. This is because the true CAR‐T cell expansions are low under scenario E4, and most patients tend to have missing measurements after the first 4 measurements.

#### 4.6.2 Correlated Toxicity and Efficacy

To demonstrate the robustness of the proposed model to correlated toxicity and efficacy endpoints, additional simulation studies are conducted to assess the operating characteristics of the proposed model under scenarios where toxicity and efficacy are positively correlated. This is under the rationale that stronger CAR‐T cells expansion drives more cytokine release [34], and hence higher toxicity. Two cases have been examined: (1) under complete measurements and (2) under MAR missing mechanism.

For the simulated pseudo data, the correlation between toxicity and efficacy is introduced using a Copula structure, as suggested in Bekele and Shen [14]. Explicitly, the toxicity and efficacy endpoints are simulated using the following steps: for patient i=1,…,n,

1. For $z^{T}\in \mathbb{R}$ and $z^{E}\in {\mathbb{R}}^{K}$, simulate ${\left(z^{T},{\left(z^{E}\right)}^{\prime }\right)}^{\prime }∼N\left(0,\sum_{\mathrm{TE}}\right)$ where $0={\left(0,\ldots ,0\right)}^{\prime }\in {\mathbb{R}}^{K+1}$, and $\sum_{\mathrm{TE}}=\left(\begin{array}{llllll} 1 & \rho_{\mathrm{TE}} & \rho_{\mathrm{TE}}^{2} & \rho_{\mathrm{TE}}^{3} & \cdots & \rho_{\mathrm{TE}}^{K}\\ \rho_{\mathrm{TE}} & 1 & \rho_{E} & \rho_{E}^{2} & \cdots & \rho_{E}^{K-1}\\ \rho_{\mathrm{TE}}^{2} & \rho_{E} & 1 & \rho_{E}^{2} & \cdots & \rho_{E}^{K-2}\\ \vdots & \vdots & \vdots & \vdots & \ddots & \vdots \\ \rho_{\mathrm{TE}}^{K} & \rho_{E}^{K-1} & \rho_{E}^{K-2} & \rho_{E}^{K-3} & \cdots & 1 \end{array}\right)\in {\mathbb{R}}^{\left(K+1\right)\times \left(K+1\right)}$. In words, the correlation between toxicity and the kth measurement of CAR‐T cells expansion is $\rho_{\mathrm{TE}}^{k}$, k=1,…,K. Among the CAR‐T cells expansions, their correlation follows an autoregression structure. The correlation between the kth and lth measurements is $\rho_{E}^{|k-l|}$, k,l=1,…,K.
2. Calculate the toxicity profile $u^{T}=\Phi \left(z^{T}\right)$ and the efficacy profile $u^{E}={\left(u_{1}^{E},\cdots ,u_{K}^{E}\right)}^{\prime }$ where $u_{k}^{E}=\Phi \left(z_{k}^{E}\right)$, k=1,…,K, where Φ(⋅) denotes the c.d.f. of the standard normal distribution N(0,1).
3. Suppose the ith patient is assigned to dose $d_{\left[i\right]}=d_{j}$, they experience a DLT if $u^{T}\leq p_{j}$, where $p_{j}$ is the marginal toxicity probabilities of dose $d_{j}$, j=1,…,J.
4. Their CAR‐T cells expansion at the kth measurement is calculated as $Z_{i,k}=F_{j,k}^{-1}\left(u_{k}^{E}\right)$, where $F_{j,k}^{-1}$ is the inverse c.d.f. of the true distribution of CAR‐T cell expansions of $d_{j}$.

##### 4.6.2.1 Complete Measurements

Assume no missing measurement to start with. The same setting as in Section 4.1 will be used. The two toxicity scenarios $T_{1}$ and $T_{2}$ will be used as the marginal toxicity distribution $p_{j}$, the five efficacy scenarios $E_{1}$–$E_{5}$ will be used as the marginal efficacy distribution $F_{j,k}$, and the correlation between them is introduced as above. The true values of the correlation coefficients are set to $\rho_{\mathrm{TE}}=0.5$ and $\rho_{E}=0.3$. These together give another 10 simulation scenarios, which will be referred to as correlated scenarios. The operating characteristics of the proposed model under these 10 correlated scenarios are compared with the 10 independent scenarios in Section 4.3. The aim is to show that, although the proposed design does not model the correlation between toxicity and efficacy, it performs equally well under correlated scenarios.

**FIGURE 10:**
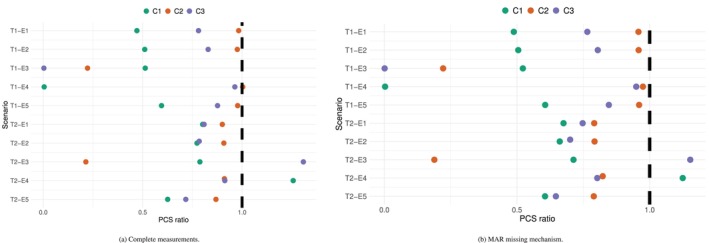
The ratio between the PCS under the proposed design and that under the non‐parametric benchmark with correlated toxicity and efficacy endpoints.

$10^{4}$ simulations have been done under each scenario, the ratio between the PCS under the proposed design and that under the non‐parametric benchmark are plotted in Figure 10 (Panel A), and the detailed proportion of recommending each dose as the OD are summarised in Table S2. Compared to the results under independent scenarios (Table 2), all the proportions are within 3% differences. This demonstrates the robustness of the proposed model towards the correlation between toxicity and efficacy without missing measurement.

##### 4.6.2.2 MAR Missing Mechanism

However, the correlation between toxicity and efficacy may induce bias in the event of MAR (missing at random) data. Consider a case where the correlation between the endpoints is combined with an informative drop out. Explicitly, patients with CRS with drop off‐study and have no further measurements for CAR‐T cell expansions. The same 10 scenarios will be used, and the correlation stays at $\rho_{\mathrm{TE}}=0.5$. The timing of the CRS is randomly simulated using a Gamma distribution, Gamma(4,2/7), with mean 14 days and standard deviation 7 days. This is based on the clinical understanding that the CRS usually appear within the first 3 weeks upon infusion.

The proportion of selecting each dose is shown in Table S3, and the ratios of PCS between the proposed design and the non‐parametric benchmark are plotted in Figure 10 (Panel B). Compared to the results with no missing measurement (Table S2), the PCS under toxicity scenario T1 is similar. This is mainly because all doses are safe under T1, and thus there are very few missing data. However, under T2, the PCS under all 5 efficacy scenarios and all 3 criteria have dropped by 5%–10%. In particular, criterion criterion $C^{\left(1\right)}$ seems to be the least affected. This is not surprising, as the CRS happens at around 2 weeks for patients with a toxicity, and thus the proportion of missingness is low around $T_{\text{threshold}}$. On the other hand, accurate estimates of $C^{\left(2\right)}$ and $C^{\left(3\right)}$ require data for the whole 14 weeks periods, and thus are more affected by the informative drop‐outs.

#### 4.6.3 Increased Noise Level

**FIGURE 11:**
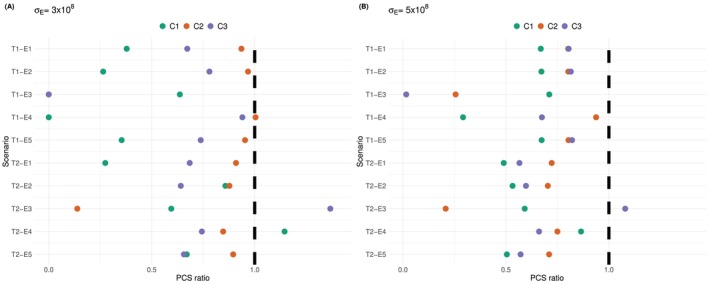
The ratio between the PCS under increase noise level $\sigma_{E}=3\times 10^{8}$ (Panel A) and $\sigma_{E}=5\times 10^{8}$ (Panel B) and the one under the original noise level $\sigma_{E}=1\times 10^{8}$. Colours correspond to the 3 efficacy criteria. Sample size n=10, based on $10^{4}$ simulations.

This section assesses the impact of changing the noise level $\sigma_{E}$. In Figure 5, $\sigma_{E}=1\times 10^{8}$ has been used. We look at increasing $\sigma_{E}$ to $3,5\times 10^{8}$. Evenly spaced measurement times once per week are assumed and all other settings stay the same as in Section 4.1. The eight scenarios are as in Table 1. The ratios of the PCS using the proposed design to the non‐parametric benchmark are plotted in Figure 11 (and detailed proportions of selecting each dose are given in the Supporting Informations).

Compared to Figure 5 where the noise level was $\sigma_{E}=1\times 10^{8}$ cells per litre, the performance under escalation criterion (C1) and (C3) improves in most scenarios, the ratio of PCS generally increases by 0.05. This is mostly because the non‐parametric benchmark is affected by the increased noise more than the proposed design. Scenario T1‐E4 and T1‐E3 remain problematic for (C1) and (C3), respectively, for the same reasons explained in Section 4.5. Under criterion (C2), the PCS ratios stays the same as $\sigma_{E}=1\times 10^{8}$ except for scenario T1‐E3, where the PCS ratio drops from 0.2 to 0, and the actual PCS drops from 18% to 0%.

Further increasing the noise level to $\sigma_{E}=5\times 10^{8}$ cells per litre (Panel B) leads to similar performance compared to $\sigma_{E}=3\times 10^{8}$ (Panel A). The PCS ratios are generally within 0.2 difference. In particular, under scenario T1‐E4 and criterion (C1), the PCS increases from close to 0% to 25%. Under scenario T1‐E3 and criterion (C2), the PCS also increases to 25%, which is higher than 18% when $\sigma_{E}=1\times 10^{8}$ and 0.3% when $\sigma_{E}=3\times 10^{8}$. The reason of the improved performance in both cases is that the increased noise makes the estimates less precise and allows exploring all four doses.

## 5 Conclusions

In conclusion, despite the fast development of CAR‐T cell therapies, appropriate early phase designs for CAR‐T cells have not been investigated before. This manuscript proposed a dose‐finding design for CAR‐T cells that uses both toxicity and efficacy information. The latter is characterised by CAR‐T cell expansions, which provide a more direct measure for treatment activity than traditional clinical response.

A bi‐exponential model has been proposed, and three possible escalation criteria have been suggested, based on the CAR‐T cells expansion at a certain time, the duration of positive expansion, or the area under the expansion trajectory. The operating characteristics of the proposed design have been compared to a non‐parametric benchmark, which provides upper bounds on the PCS. Overall, the proposed model demonstrates good performance under a wide range of toxicity and efficacy scenarios, and robustness towards missing measurements, correlated toxicity and efficacy endpoints, and increased noise levels.

However, the proposed model has the following limitations. Firstly, the model is designed to capture only the mean CAR‐T cells expansion on the population level, the patient‐specific effect cannot be estimated. Nevertheless, since the aim of the trial is to identify the recommended dose on the population level, predicting the CAR‐T cells trajectory for each individual patient is not necessary. Secondly, under criterion $C^{\left(2\right)}$, the choice of the minimal effective level $Z_{\text{threshold}}$ needs to be carefully discussed with the clinical team. The simulation studies show that if $Z_{\text{threshold}}$ is very close to the lowest dose level, and a large proportion of patients are believed not to have the expansion period of CAR‐T cells, the performance of the bi‐exponential model can be undermined. In that case, escalation should be based on the other criteria. Moreover, by construction, the bi‐exponential model can only have at most one expansion period. If the data suggests a second expansion, this cannot be captured by the model.

The CRM has been used to model the dose‐toxicity relationship. However, when patients are not allocated to the best estimate of the MTD, the design is not well adapted.

The proposed design assumed independence between efficacy and toxicity given the data. Sensitivity analysis showed that not modelling the correlation has little impact on the point estimates of the toxicity probabilities and the CAR‐T cell expansions, even under strongly correlated simulated data. On the other hand, under the MAR missing mechanism, when the missing CAR‐T cell expansion measurement is due to an early drop‐out following a DLT outcome, the proposed model would potentially lead to biassed estimates. For CAR‐T cells, this is not of big concern, since the toxicities are very low, and the DLT rate remains low. Nevertheless, this is an issue that has been largely overlooked by most of the early phase trial designs. In order to deal with this potential bias, a joint modelling of the toxicity and efficacy outcomes is required, which requires further work.

Furthermore, the model cannot handle irregular measurement times if they are taken on non‐overlapping days. In reality, this can happen because measurements are taken on the same day of the week regardless of the infusion day. Solving this irregular measurement problem remains future work.

Note that the proposed model does not account for the potential two subpopulations among patients, and thus cannot be used to categorise patients into subpopulations. This could be looked at as a future work in the context of random effect models and latent class model. This information could potentially help to better characterise the individual trajectories if of interest.

The current expansion trajectory can only have one expansion period, whereas a potential second expansion might appear as observed on real data. Cell therapy is an extremely promising field of therapeutic progress and dose optimisation needs to rely on adequate models of activity and toxicity.

## Funding

This report is independent research supported by the National Institute for Health Research (NIHR300576). The views expressed in this publication are those of the authors and not necessarily those of the NHS, the National Institute for Health Research or the Department of Health and Social Care (DHSC). PM also received funding from UK Medical Research Council (MC_UU_00040/3). For the purpose of open access, the author has applied a Creative Commons Attribution (CC BY) licence to any Author Accepted Manuscript version arising. WC receives the Gates Cambridge Scholarship for her PhD in Biostatistics. XP receives support from ITMO Cancer of Aviesan on funds administered by INSERM ANR‐21‐RHUS‐0016 (RHU‐EpCART).

## Ethics Statement

The authors have nothing to report.

## Conflicts of Interest

The authors declare no conflicts of interest.

## Supporting information


**Table S1:** The proportion of selecting each dose under the proposed design and the non‐parametric benchmark when changing the threshold Z_threshold_. The ODs are highlighted in bold. All estimates are based on 10^4^ simulations.
**Table S2:** The proportion of selecting each dose as the OD under 10 scenarios and 3 criteria with correlated toxicity and efficacy endpoints. The PCS are highlighted in **bold**.
**Table S3:** The proportion of selecting each dose as the OD under 10 scenarios and 3 criteria with correlated toxicity and efficacy endpoints and MAR missing mechanism. The PCS are highlighted in **bold**.
**Table S4:** The proportion of selecting each dose by the proposed design under the 10 independent scenarios, under noise level *σ* = 3,5 × 10^8^ cell sperlitre. The ODs are highlighted in bold. All estimates are based on 10^4^ simulations.
**Table S5:** The proportion of selecting each dose as the OD with sample size *n* = 20. (a): Results under independent toxicity and efficacy endpoints. (b): Results under correlated toxicity and efficacy endpoints with *ρ*_*TE*_ = 0.5. The PCS are highlighted in **bold**.
**Figure S1:** The ratio between PCS under the proposed design and under the non‐parametric benchmark with sample size *n* = 20.
**Table S6:** The proportion of negative simulated cell count under 10 scenarios, based on 10^4^ simulations
**Table S7:** The proportion of selecting each dose as the OD under 10 scenarios and 3 criteria with starting dose *d*_2_. The PCS are highlighted in **bold**.
**Figure S2:** The ratio between PCS under the proposed design and the non‐parametric benchmark with sample size *n* = 10 and starting dose *d*_2_.
**Table S8:** The proportion of selecting each dose as the OD under 10 scenarios and 3 criteria with starting dose *d*_2_. The PCS are highlighted in **bold**.
**Figure S3:** Theratio between PCS under the proposed design and under the non‐parametric benchmark with 5% (Panel A) and 15% (Panel B) equivalence margins.

## Data Availability

Only simulated data has been used. The R code can be found at https://github.com/WeishiC/CAR‐T‐cells.
