# Supplementary material for: Early Phase Dose‐Finding Designs for CAR‐T Cell Therapies
Source: Pharm Stat. 2026 Jun 18;25(4):e70102. doi: 10.1002/pst.70102 (PMC13277398; doi:10.1002/pst.70102)
Supplement: Supplementary file 1 — Table S1: The proportion of selecting each dose under the proposed design and the non‐parametric benchmark when changing the threshold Zthreshold. The ODs are highlighted in bold. All estimates are based on 104 simulations. Table S2: The proportion of selecting each dose as the OD under 10 scenarios and 3 criteria with correlated toxicity and efficacy endpoints. The PCS are highlighted in bold. Table S3: The proportion of selecting each dose as the OD under 10 scenarios and 3 criteria with correlated toxicity and efficacy endpoints and MAR missing mechanism. The PCS are highlighted in bold. Table S4: The proportion of selecting each dose by the proposed design under the 10 independent scenarios, under noise level σ = 3,5 × 10^8^ cell sperlitre. The ODs are highlighted in bold. All estimates are based on 104 simulations. Table S5: The proportion of selecting each dose as the OD with sample size n = 20. (a): Results under independent toxicity and efficacy endpoints. (b): Results under correlated toxicity and efficacy endpoints with ρ TE = 0.5. The PCS are highlighted in bold. Figure S1: The ratio between PCS under the proposed design and under the non‐parametric benchmark with sample size n = 20. Table S6: The proportion of negative simulated cell count under 10 scenarios, based on 104 simulations Table S7: The proportion of selecting each dose as the OD under 10 scenarios and 3 criteria with starting dose d 2. The PCS are highlighted in bold. Figure S2: The ratio between PCS under the proposed design and the non‐parametric benchmark with sample size n = 10 and starting dose d 2. Table S8: The proportion of selecting each dose as the OD under 10 scenarios and 3 criteria with starting dose d 2. The PCS are highlighted in bold. Figure S3: Theratio between PCS under the proposed design and under the non‐parametric benchmark with 5% (Panel A) and 15% (Panel B) equivalence margins. [file PST-25-0-s001.pdf]

Early Phase Dose-Finding Designs for CAR-T cell Therapies Supplementary Materials

S1 | THE GROWTH MODEL FOR CAR-T CELL EXPANSIONS

Let $\tilde{X}_{i,t}, \tilde{Y}_{i,t}$ be the number of effective and exhaustive cells for patient i, $i = 1, \ldots, n$, at time $t \geq 0$, respectively, and let $\tilde{Z}_{i,t} = \tilde{X}_{i,t} + \tilde{Y}_{i,t}$ be the total number of CAR-T cells. Let $d_j, j = 1, \ldots, J$, be the dose levels being investigated and $d_{[i]}$ denotes the dose level received by patient i, $d_{[i]} \in \{d_1, \ldots, d_J\}$. Then, the dynamic can be described by the set of differential equations

$$\begin{aligned} \frac{d\tilde{X}_{i,t}}{dt} &= \tilde{b}_1 \tilde{X}_{i,t} \times d_{[i]} - \tilde{b}_2 \tilde{Y}_{i,t} \times d_{[i]} \\ \frac{d\tilde{Y}_{i,t}}{dt} &= \tilde{c}_1 \tilde{X}_{i,t} - \tilde{c}_2. \end{aligned}, \tag{S1}$$

where $\tilde{b}_1, \tilde{b}_2$ describes the rate of expansion and exhaustion among effective cells, and $\tilde{c}_1, \tilde{c}_2$ describes the rate of exhaustion and elimination among exhaustive cells, respectively.

Model (S1) can be considered as a simplification of the models proposed in Paixao et al (2022)[1], which will be referred to as the *Paixao model*. More explicitly, they considered 4 types of CAR-T cells – distributed (C_D), effector (C_T), memory (C_M), and exhausted (C_E), which are graphically shown in their Figure 1(b). Since their original 18-parameter model cannot be reliably estimated based on 10 patients in our setting, we make the following simplifications.

- Omit the initial distribution phase: the difference between the number of cells infused and engrafted has been ignored, and thus C_D is omitted from the system.
- Integrate together the effector and memory cells, and call them both effective cells (which we denote as $\tilde{X}_{i,t}$ for patient i at time t). Assume a constant expansion rate for these effective cells (which we denote as $\tilde{b}_1$).
- Integrate together the rates of inhibition due to interaction with tumour cells and exhaustion, and assume a constant rate (which we denote as $\tilde{b}_2$).

The ODE for exhausted cells, which we denoted as $\tilde{Y}_{i,t}$ for patient i at time t, is the same as that in the Paixao model, and our notations for the exhaustion and death rates are $\tilde{c}_1$ and $\tilde{c}_2$, respectively.

Due to this similarities between Model (S1) and the Paixao model, we expect the solutions to be approximately the same. In particular, let $\tilde{Z}_{i,t} = \tilde{X}_{i,t} + \tilde{Y}_{i,t}$ be the total number of CAR-T cells, it has been derived in Paixao et al (2022) that the expansion phase can be approximated by

$$\frac{d\tilde{Z}_{i,t}}{d_t} \approx \tilde{b}_1 d_{[i]} \tilde{Z}_{i,t},$$

leading to the solution $d_{[i]} \exp\{\tilde{b}_1 d_{[i]} t\}$ under the initial condition $\tilde{Z}_{i,0} = d_{[i]}$. Similarly, the contraction phase can be approximated by

$$\frac{d\tilde{Z}_{i,t}}{d_t} \approx -\tilde{c}_2 d_{[i]} \tilde{Z}_{i,t},$$

leading to the solution $d_{[i]} \exp\{-\tilde{c}_2 d_{[i]} t\}$ under the same initial condition. This motivates the two exponential parts in our bi-exponential model.

Figure 3 in Paixao et al (2022) gave some trajectories fitted under their 18-parameter model, and we see that the general shapes of the trajectories do look similar to the outputs from our bi-exponential model.

S2 | CHANGE THE THRESHOLDS

The proposed design was evaluated under $Z_{\text{threshold}} = 0.25 \times 10^8$ cells per litre in Section 4.5 in the main text, Table S1 below shows the result when increasing $Z_{\text{threshold}}$ from 0.25 to 0.3×10^8 or decreasing to 0.2×10^8 cells per litre.

Scenario	Proposed design				Benchmark			
	d_1	d_2	d_3	d_4	d_1	d_2	d_3	d_4
T1-E1	**97.33**	0	0	0	**96.35**	3.65	0	0
T1-E2	**98.30**	0	0	0	**96.00**	4	0	0
T1-E3	35.44	**56.80**	0	0	0.25	**99.75**	0	0
T1-E4	**91.50**	4.13	0	0	**71.55**	28.45	0	0
T1-E5	**94.53**	0	0	0	**96.35**	3.65	0	0
T2-E1	**90.05**	0	0	0	**95.80**	3.75	0	0
T2-E2	**87.38**	0	0	0	**95.40**	4.15	0	0
T2-E3	32.28	**44.90**	0	0	4.05	**95.50**	0	0
T2-E4	**77.91**	2.43	0	0	**72.45**	27	0	0
T2-E5	**89.76**	0.2	0	0	**95.80**	3.75	0	0

(a) $Z_{\text{threshold}} = 0.3 \times 10^8$.

Scenario	Proposed design				Benchmark			
	d_1	d_2	d_3	d_4	d_1	d_2	d_3	d_4
T1-E1	**97.19**	0	0	0	**100**	0	0	0
T1-E2	**95.58**	0	0	0	**100**	0	0	0
T1-E3	**98.39**	0.4	0	0	**89.10**	10.9	0	0
T1-E4	**99.20**	0	0	0	**100**	0	0	0
T1-E5	**96.22**	0	0	0	**100**	0	0	0
T2-E1	**88.35**	0	0	0	**99.47**	0	0	0
T2-E2	**88.76**	0	0	0	**99.60**	0	0	0
T2-E3	**87.15**	0.8	0	0	**89.23**	10.2	0	0
T2-E4	**89.56**	0	0	0	**99.63**	0	0	0
T2-E5	**90.02**	0	0	0	**99.47**	0	0	0

(b) $Z_{\text{threshold}} = 0.2 \times 10^8$.

TABLE S1 The proportion of selecting each dose under the proposed design and the non-parametric benchmark when changing the threshold $Z_{\text{threshold}}$. The ODs are highlighted in bold. All estimates are based on 10^4 simulations.

S3 | CORRELATED SCENARIOS

Table S2 gives the proportion of selecting each dose when the toxicity and efficacy endpoints are simulated with positive correlation $\rho_{TE} = 0.5$. Assume complete measurements of all endpoints.

Scenario	Dose levels				Dose levels				Dose levels			
	d_1	d_2	d_3	d_4	d_1	d_2	d_3	d_4	d_1	d_2	d_3	d_4
	$C^{(1)}$				$C^{(2)}$				$C^{(3)}$			
T1-E1	2.23	24.31	32.72	**39.42**	**98.29**	0	0	0	3.15	19.45	**74.77**	1.31
T1-E2	1.31	23.13	32.06	**42.31**	**97.50**	0	0	0	3.55	17.08	**76.87**	1.45
T1-E3	1.58	19.58	35.35	**42.31**	74.38	**18.40**	0	0	3.94	13.01	81.47	**0.26**
T1-E4	2.76	95.40	0	**0.39**	**98.42**	0	0	0	2.89	**95.01**	0.13	0
T1-E5	2.50	24.84	29.57	**41.52**	**97.77**	0	0	0	3.81	18.40	**69.51**	7.1
T2-E1	5.65	41.39	**33.11**	9.99	**89.75**	0	0	0	11.30	26.68	**53.88**	0.66
T2-E2	6.18	41.79	**31.67**	12.48	**90.01**	0	0	0	9.46	30.09	**53.22**	0.39
T2-E3	6.57	40.34	**33.64**	12.48	66.62	**17.08**	0	0	10.51	23.92	**58.48**	0
T2-E4	7.62	**84.10**	0	0	**88.30**	0	0	0	7.23	**85.02**	0	0
T2-E5	7.36	41.00	**28.78**	14.59	**86.60**	0	0	0	9.33	32.33	**47.17**	3.94

TABLE S2 The proportion of selecting each dose as the OD under 10 scenarios and 3 criteria with correlated toxicity and efficacy endpoints. The PCS are highlighted in **bold**.

Table S3 combines the correlated toxicity and efficacy endpoints with the MAR missing mechanism. Patients will drop-out when a CRS is observed, and thus no further efficacy measurement is available.

Scenario	Dose levels				Dose levels				Dose levels			
	d_1	d_2	d_3	d_4	d_1	d_2	d_3	d_4	d_1	d_2	d_3	d_4
	$C^{(1)}$				$C^{(2)}$				$C^{(3)}$			
T1-E1	1.41	22.36	31.5	**40.84**	**95.78**	0	0	0	3.69	18.22	**73.25**	1.32
T1-E2	2.26	21.21	30.94	**41.84**	**95.75**	0	0	0	3.08	17.67	**74.57**	1.29
T1-E3	1.82	19.89	31.85	**43.07**	73.13	**18.31**	0	0	3.46	16.03	77.29	**0.09**
T1-E4	2.52	93.17	0.06	**0.23**	**95.61**	0	0	0	2.55	**93.61**	0.21	0.23
T1-E5	1.73	21.36	31.06	**42.31**	**96.02**	0	0	0	2.7	18.93	**67.10**	7.94
T2-E1	5.65	39.32	**27.89**	9.96	**78.82**	0	0	0	8.64	23.79	**49.78**	0.32
T2-E2	5.63	39.2	**27.07**	10.52	**78.52**	0	0	0	9.96	24.99	**47.52**	0.38
T2-E3	6.12	36.04	**30.44**	11.31	59.42	**15.03**	0	0	9.43	21.36	**51.54**	0.15
T2-E4	6.83	**75.24**	0.09	0.15	**79.75**	0.03	0	0	6.45	**74.71**	0.09	0.03
T2-E5	5.27	38.91	**27.86**	9.49	**78.70**	0	0	0	7.88	28.16	**42.54**	3.08

TABLE S3 The proportion of selecting each dose as the OD under 10 scenarios and 3 criteria with correlated toxicity and efficacy endpoints and MAR missing mechanism. The PCS are highlighted in **bold**.

S4 | INCREASING THE NOISE LEVEL

In the setting described in Section 4.6.2, the proportion of selecting each dose with noise level $\sigma_E = 3, 5 \times 10^8$ cells per litre are summarised in Table S4 below.

Scenario	$C^{(1)}$				$C^{(2)}$				$C^{(3)}$			
	d_1	d_2	d_3	d_4	d_1	d_2	d_3	d_4	d_1	d_2	d_3	d_4
	Proposed design, $\sigma_E = 3 \times 10^8$											
T1-E1	3.1	25.0	37.5	**34.4**	**93.8**	0	0	0	3.1	25.0	**65.6**	6.3
T1-E2	0	28.1	50.0	**21.9**	**96.9**	0	0	0	6.3	12.5	**71.9**	6.3
T1-E3	0	18.8	21.9	**56.3**	71.9	**3.1**	0	0	3.1	6.3	84.4	**0**
T1-E4	6.3	93.8	0	**0**	**100**	0	0	0	6.3	**93.8**	0	0
T1-E5	3.0	25.2	37.3	**34.5**	**92.5**	1.3	0	0	3.4	25.0	**65.7**	6.5
T2-E1	0	71.9	**12.5**	9.4	**90.6**	0	0	0	0	37.5	**46.9**	6.3
T2-E2	0	37.5	**40.6**	9.4	**87.5**	0	0	0	9.4	34.4	**46.9**	3.1
T2-E3	6.3	50.0	**28.1**	15.6	71.9	**15.6**	0	0	12.5	21.9	**65.6**	0
T2-E4	6.3	**84.4**	0	0	**84.4**	0	0	0	9.4	**71.9**	3.1	0
T2-E5	1.2	68.8	**21.2**	8.8	**92.4**	0	0	0	0	34.5	**48.1**	17.4

(a) $\sigma_E = 3 \times 10^8$.

Scenario	$C^{(1)}$				$C^{(2)}$				$C^{(3)}$			
	d_1	d_2	d_3	d_4	d_1	d_2	d_3	d_4	d_1	d_2	d_3	d_4
T1-E1	1.1	15.7	23.5	**57.8**	**80.2**	0.3	16.3	0	1.8	12.5	**77.0**	6.4
T1-E2	1.6	15.3	24.3	**57.1**	**80.2**	0.1	17.2	0	2.2	12.7	**78.0**	5.5
T1-E3	1.3	13.3	24.0	**59.8**	69.6	**24.7**	1.3	0	2.2	11.4	83.5	**1.3**
T1-E4	2	60.6	10.1	**24.9**	**91.3**	2.3	3.6	0.1	1.9	**67.2**	28.7	0.2
T1-E5	1.1	15.8	23.8	**57.4**	**80.4**	0.4	16.1	0	1.9	12.4	**76.6**	6.8
T2-E1	5.3	37.0	**23.4**	14.8	**71.8**	0.3	8.3	0	9.5	31.0	**39.7**	2.3
T2-E2	7.4	36.2	**24.7**	13.8	**70.0**	0.1	8.3	0	9.3	27.5	**42.7**	2.1
T2-E3	8.1	28.6	**29.5**	16.9	59.9	**19.2**	1.2	0	9.7	24.3	**50.5**	0.5
T2-E4	7.3	**63.4**	4.2	7.0	**73.0**	1.8	3.9	0	7.8	**63.5**	8.2	0
T2-E5	5.5	36.0	**23.9**	15.1	**72.1**	0.3	7.8	0	9.5	30.2	**40.1**	1.3

(b) $\sigma_E = 5 \times 10^8$.

TABLE S4 The proportion of selecting each dose by the proposed design under the 10 independent scenarios, under noise level $\sigma = 3, 5 \times 10^8$ cells per litre. The ODs are highlighted in bold. All estimates are based on 10^4 simulations.

S5 | SAMPLE SIZES

The proportion of selecting each dose as the OD at sample size $n = 20$ under 20 scenarios (10 independent scenarios and 10 correlated scenarios with $\rho_{TE} = 0.5$) are shown in Table S5 below, all based on 10^4 simulations. The ratios of PCS under the proposed design and the non-parametric benchmark is shown in Figure S1.

Scenario	Dose levels				Dose levels				Dose levels			
	d_1	d_2	d_3	d_4	d_1	d_2	d_3	d_4	d_1	d_2	d_3	d_4
	$C^{(1)}$				$C^{(2)}$				$C^{(3)}$			
T1-E1	0	3.65	17.86	**76.32**	**97.56**	0	0	0	0.27	3.79	**93.78**	0.14
T1-E2	0	3.52	20.57	**74.29**	**97.97**	0	0	0	0.14	3.92	**93.78**	0.14
T1-E3	0	3.65	18.00	**77.40**	81.46	**11.37**	0	0	0	5.95	93.10	**0**
T1-E4	0.14	96.89	0	**1.08**	**97.43**	0	0	0	0.14	**98.11**	0	0.27
T1-E5	0	4.47	19.89	**72.94**	**97.02**	0	0	0	0	3.25	**89.85**	5.95
T2-E1	2.57	22.19	**39.38**	27.33	**89.72**	0	0	0	1.89	20.03	**69.01**	0.14
T2-E2	1.89	21.24	**42.63**	26.93	**88.23**	0	0	0	1.49	20.84	**68.74**	0
T2-E3	1.76	23.68	**42.35**	25.03	77.00	**8.53**	0	0	1.35	22.33	**68.88**	0
T2-E4	1.22	**89.04**	0.41	0.68	**87.28**	0	0	0	1.49	**87.55**	0	0
T2-E5	1.62	20.97	**42.76**	26.39	**88.09**	0	0	0	1.62	19.22	**68.06**	2.84

(a) Independent toxicity and efficacy endpoints.

Scenario	Dose levels				Dose levels				Dose levels			
	d_1	d_2	d_3	d_4	d_1	d_2	d_3	d_4	d_1	d_2	d_3	d_4
	$C^{(1)}$				$C^{(2)}$				$C^{(3)}$			
T1-E1	0.13	3.60	18.13	**76.40**	**98.13**	0	0	0	0.13	4.40	**93.20**	0.13
T1-E2	0.13	3.33	17.87	**77.47**	**97.33**	0	0	0	0.13	3.33	**95.20**	0.13
T1-E3	0	3.73	19.20	**75.20**	85.20	**8.93**	0	0	0.13	4.67	94.27	**0**
T1-E4	0	97.33	0.13	**0.53**	**98.67**	0	0	0	0.67	**97.60**	0	0.13
T1-E5	0	3.47	18.53	**76.13**	**98.13**	0	0	0	0	3.60	**88.13**	6.53
T2-E1	2.27	22.93	**43.60**	23.47	**87.20**	0	0	0	2.00	23.87	**65.73**	0
T2-E2	1.73	20.67	**42.80**	24.13	**88.53**	0	0	0	2.13	18.67	**71.60**	0
T2-E3	2.27	23.07	**41.87**	25.07	75.73	**7.87**	0	0	2.40	18.93	**70.53**	0
T2-E4	0.80	**90.40**	0.27	0.40	**88.40**	0	0	0	0.93	**91.60**	0	0.13
T2-E5	1.87	22.40	**43.20**	23.87	**88.80**	0	0	0	2.27	25.20	**62.67**	1.87

(b) Correlated toxicity and efficacy endpoints.

TABLE S5 The proportion of selecting each dose as the OD with sample size $n = 20$. (a): Results under independent toxicity and efficacy endpoints. (b): Results under correlated toxicity and efficacy endpoints with $\rho_{TE} = 0.5$. The PCS are highlighted in **bold**.

Compare to the results under $n = 10$, all the problematic scenarios (scenario T1-E3 under $C^{(3)}$ due to over-estimating the toxicity of d_4; scenario T1-E4 under $C^{(1)}$ due to the observed data having two expansions; scenario E3 under $C^{(2)}$ due to setting $Z_{\text{threshold}} = d_1$) remain to have low PCS. In particular, under scenario T1-E3 and criterion $C^{(3)}$, the problem of not assigning patients to the best estimates of the MTD remains. This problem cannot be mitigated by the larger sample size.

On the other hand, all the other scenarios have operating characteristics improved substantially, most of the PCS ratios to the benchmark are now close to 1, as shown in Figure S1. In particular, the PCS under scenario T1 and criterion $C^{(1)}$, when the OD is d_4, has increased by around 30%. This is because a larger sample sizes allows a more sufficient exploration of higher doses.

S6 | NEGATIVE SIMULATED CAR-T CELL EXPANSIONS

The proposed model only fit mean CAR-T cell expansions on the population level instead of the patient-specific expansions. Hence, under the constraint $b_2 \leq b_1 < 0$, the mean expansion (i.e., fitted value) is always non-negative. However, negative cell count may occur in the simulated pseudo data, and the proportions of negative counts under each scenario are summarised in Table S6 below. It can be seen that the proportion of negative counts is at most 1.3%.

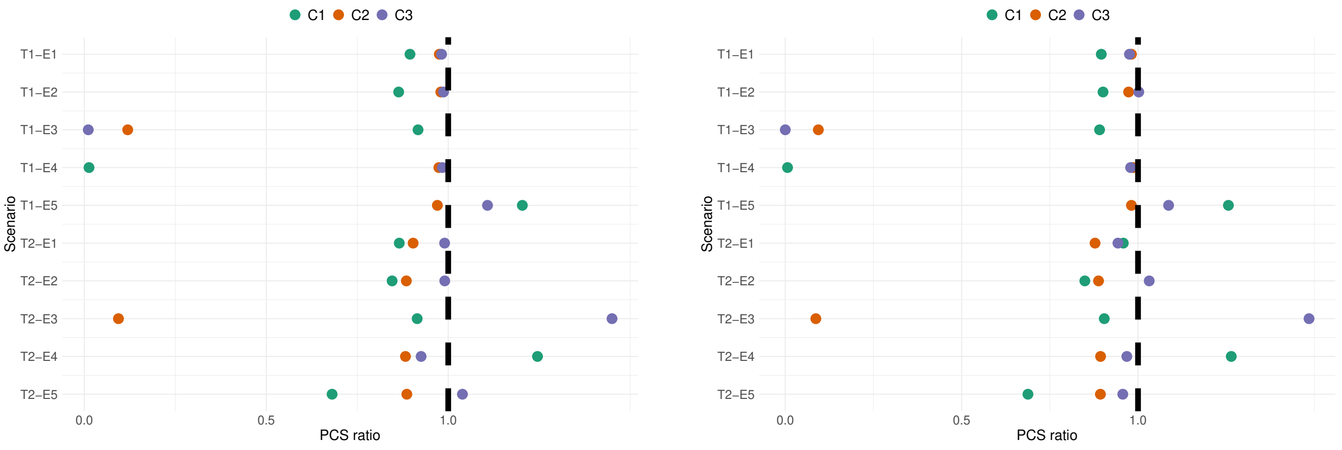

(a) Independent toxicity and efficacy endpoints.

(b) Correlated toxicity and efficacy endpoints.

FIGURE S1 The ratio between PCS under the proposed design and under the non-parametric benchmark with sample size $n = 20$.

	Efficacy scenario				
	E1	E2	E3	E4	E5
T1	0.69	0.72	1.27	0.14	0
T2	0.39	0.42	0.92	0.18	0

TABLE S6 The proportion of negative simulated cell count under 10 scenarios, based on 10^4 simulations.

S7 | STARTING DOSE

For our motivating trial, and for most Phase I dose-finding trials in general, the starting dose is a clinical decision, which conventionally would be the lowest dose levels. Nevertheless, the operating characteristics when starting at d_2 have been assessed. The simulation results with sample size $n = 10$ and independent toxicity and efficacy can be found in Table S7, and the ratio of PCS between the proposed model and the non-parametric benchmark is plotted in Figure S2.

Scenario	Dose levels				Dose levels				Dose levels			
	d_1	d_2	d_3	d_4	d_1	d_2	d_3	d_4	d_1	d_2	d_3	d_4
	$C^{(1)}$				$C^{(2)}$				$C^{(3)}$			
T1-E1	1.67	5.66	28.52	**52.58**	**89.41**	0	0	0	1.52	3.03	**83.27**	0.69
T1-E2	1.65	6.45	28.42	**51.60**	**88.93**	0	0	0	1.52	3.63	**82.73**	1.04
T1-E3	1.88	6.35	29.94	**50.16**	76.28	**11.21**	0	0	1.55	2.61	84.76	**0.17**
T1-E4	2.57	82.38	1.96	**2.55**	**88.95**	0	0	0	2.57	**83.23**	1.27	2.26
T1-E5	1.65	6.39	29.46	**51.91**	**88.68**	0	0	0	1.65	3.47	**76.34**	7.41
T2-E1	5.41	16.08	**34.43**	19.00	**72.94**	0	0	0	4.95	7.48	**62.43**	0.38
T2-E2	5.66	16.43	**33.79**	18.71	**71.35**	0	0	0	4.03	7.66	**61.56**	0.21
T2-E3	5.76	16.10	**34.56**	18.46	63.77	**10.48**	0	0	4.66	6.56	**64.61**	0.08
T2-E4	6.18	**65.71**	1.77	0.9	**71.73**	0	0	0	6.31	**66.49**	1.52	0.56
T2-E5	5.14	15.56	**34.54**	19.4	**71.54**	0	0	0	4.3	9.25	**58.28**	3.17

TABLE S7 The proportion of selecting each dose as the OD under 10 scenarios and 3 criteria with starting dose d_2. The PCS are highlighted in **bold**.

Overall, the operating characteristics between starting at d_1 and d_2 are very similar. The PCS when starting at d_2 becomes higher for scenarios where the OD is at higher doses and lower for scenarios where the OD is at d_1. Importantly, the four scenarios with very low PCS (T1-E3 under $C^{(2)}$, $C^{(3)}$, T1-E4 under $C^{(1)}$, and T2-E3 under $C^{(2)}$) remain to have poor performance. This shows that the conclusions are robust towards the starting dose.

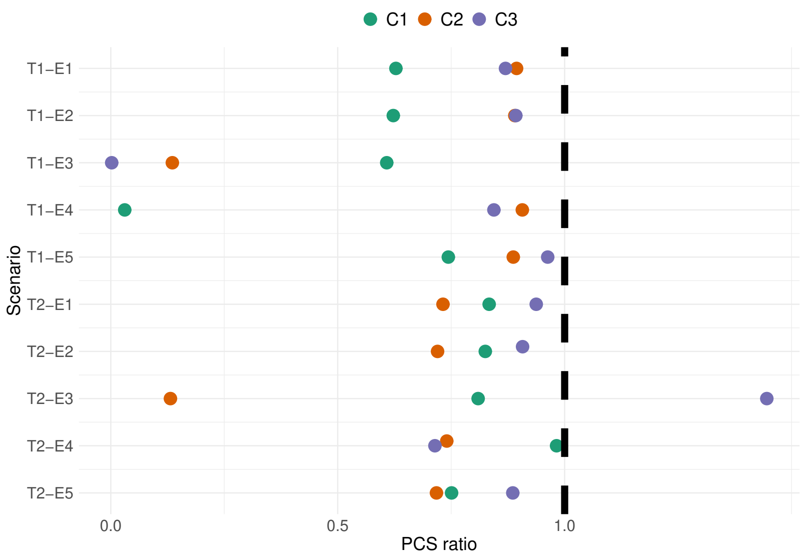

FIGURE S2 The ratio between PCS under the proposed design and the non-parametric benchmark with sample size n = 10 and starting dose d_2.

S8 | EQUIVALENCE MARGIN

The operating characteristics with 5% and 15% equivalence margins have been assessed. The simulation results can be found below in Table S8, and the PCS ratios to the non-parametric benchmark are plotted in Figure S3. Note that since the definition of the OD depends on the equivalence margin, some of the ODs with the 5% equivalence margin have changed.

Scenario	Dose levels				Dose levels				Dose levels			
	d_1	d_2	d_3	d_4	d_1	d_2	d_3	d_4	d_1	d_2	d_3	d_4
	$C^{(1)}$				$C^{(2)}$				$C^{(3)}$			
T1-E1	2.18	22.34	31.24	**41.25**	**95.75**	0	0	0	1.59	23.33	30.97	**40.82**
T1-E2	1.94	21.69	30.41	**42.41**	**95.61**	0	0	0	1.96	22.6	30.44	**41.71**
T1-E3	1.43	20.32	32.1	**42.44**	71.72	**20.59**	0	0	2.45	19.97	38.35	**35.84**
T1-E4	2.21	93.97	0	**0**	**95.99**	0	0	0	2.1	**94.03**	0	0
T1-E5	1.64	21.8	30.68	**42.38**	**95.69**	0	0	0	1.48	23.68	29.55	**41.87**
T2-E1	6.24	37.46	**27.53**	10.98	**80.41**	0	0	0	5.25	38.16	**28.07**	11.3
T2-E2	5.54	39.1	**27.74**	10.63	**79.36**	0	0	0	6.03	39.72	**26.70**	9.77
T2-E3	5.33	37.46	**30.19**	10.84	59.69	**16.17**	0	0	6.84	34.85	**31.35**	9.66
T2-E4	7.78	**74.22**	0	0.03	**79.33**	0	0	0	7.45	**75.03**	0.03	0
T2-E5	5.73	37.57	**28.31**	10.63	**80.60**	0	0	0	5.41	38.24	**27.56**	10.76

(a) 5% equivalence margin.

Scenario	Dose levels				Dose levels				Dose levels			
	d_1	d_2	d_3	d_4	d_1	d_2	d_3	d_4	d_1	d_2	d_3	d_4
	$C^{(1)}$				$C^{(2)}$				$C^{(3)}$			
T1-E1	1.9	21.74	30.61	**42.41**	**96.13**	0	0	0	3.54	16.48	**76.48**	0
T1-E2	1.46	22.09	30.95	**42.04**	**96.15**	0	0	0	4.07	16.4	**76.19**	0
T1-E3	1.6	20.28	33.26	**41.80**	77.06	**15.12**	0	0	3.24	13.18	80.51	**0**
T1-E4	2.79	93.5	0.02	**0.16**	**95.77**	0	0	0	2.31	**93.44**	0.3	0.32
T1-E5	1.62	22.53	30.22	**42.25**	**95.22**	0	0	0	3.7	17.02	**76.01**	0
T2-E1	5.89	38.2	**27.69**	10.87	**80.10**	0	0	0	10.3	23.16	**50.38**	0
T2-E2	5.22	39.78	**27.94**	10.69	**80.75**	0	0	0	9.72	23.04	**50.87**	0
T2-E3	5.81	35.36	**30.91**	11.82	63.58	**11.58**	0	0	9.23	19.47	**55.47**	0
T2-E4	6.9	**76.17**	0.08	0.06	**79.43**	0	0	0	6.34	**75.85**	0.32	0.08
T2-E5	5.26	40.00	**28.14**	10.06	**79.23**	0	0	0	9.94	22.59	**51.66**	0

(b) 15% equivalence margin.

TABLE S8 The proportion of selecting each dose as the OD under 10 scenarios and 3 criteria with starting dose d_2. The PCS are highlighted in **bold**.

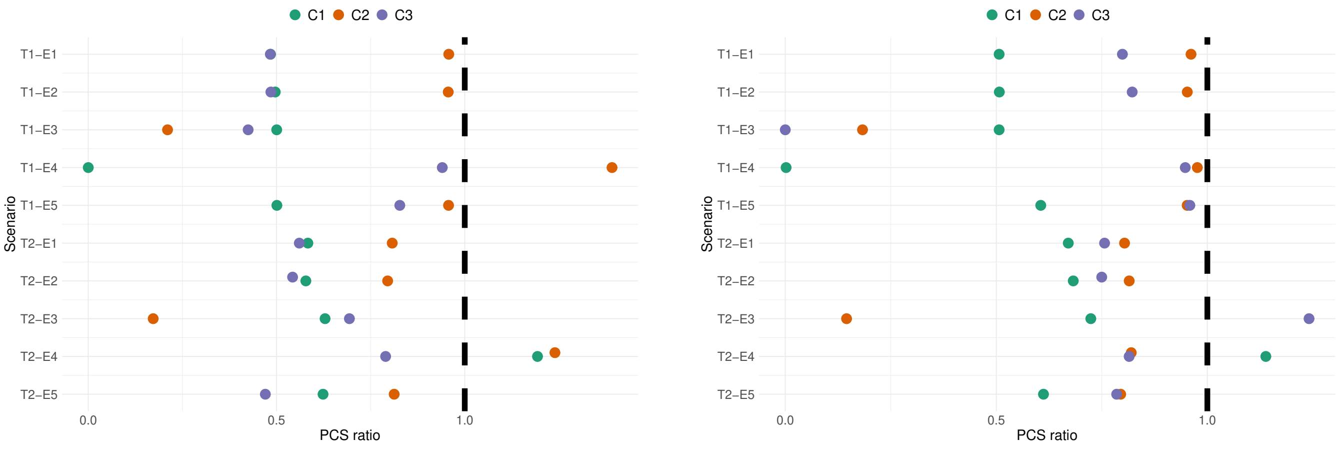

(a) 5% equivalence margin.

(b) 15% equivalence margin.

FIGURE S3 The ratio between PCS under the proposed design and under the non-parametric benchmark with 5% (Panel A) and 15% (Panel B) equivalence margins.

The results with 15% equivalence margin remains very similar to that with 10%. However, with 5% margin, the problem of scenario T1-S3 under $C^{(3)}$ has been mitigated. This makes intuitive sense, as decreasing the equivalence margin makes it harder for the AUC under lower doses to be equivalent to that under higher doses. This encourages assigning patients to higher doses, and thus the toxicity model now has the opportunity to explore higher doses and better fit the toxicity at d_4.

REFERENCES

1. Paixao EA, Barros LRC, Fassoni AC, Almeida RC. Modeling Patient-Specific CAR-T Cell Dynamics: Multiphasic Kinetics via Phenotypic Differentiation. *Cancers.* 2022;14(22):5576.
